# Facile Access to Stable Silylium Ions Stabilized by *N*-Heterocyclic Imines

**DOI:** 10.3390/molecules21091155

**Published:** 2016-08-30

**Authors:** Tatsumi Ochiai, Tibor Szilvási, Shigeyoshi Inoue

**Affiliations:** 1 Institut für Chemie, Technische Universität Berlin, Straße des 17. Juni 135, Sekr. C2, 10623 Berlin, Germany; tatsumi.ochiai@mailbox.tu-berlin.de; 2 Department of Inorganic and Analytical Chemistry, Budapest University of Technology and Economics, Szent Gellért tér 4, 1111 Budapest, Hungary; szilvasitibor@ch.bme.hu; 3 Department of Chemistry, Catalysis Research Center and Institute of Silicon Chemistry, Technische Universität München, Lichtenbergstraße 4, 85748 Garching, Germany

**Keywords:** silylium, imine, cation, silicon, borate

## Abstract

Novel silylium ions with *N*-heterocyclic imines were successfully synthesized. The reaction of trimethylsilyl imidazolin-2-imine Me_3_SiNIPr (NIPr = bis(2,6-diisopropylphenyl)-imidazolin-2-imino) with B(C_6_F_5_)_3_ leads to dimeric imino-substituted silylium ions through a methyl group abstraction on the silicon atom. Meanwhile, the intermolecular imino-coordinated silylium ion is formed by using the less sterically crowded imine Me_3_SiNI*t*Bu (NI*t*Bu = bis(*tert*-butyl)-imidazolin-2-imino). Furthermore, the treatment of dimethylchlorosilane Me_2_(Cl)SiNIPr with AgOTf affords the contact ion pair Me_2_(OTf)SiNIPr by substitution of the chloride. A novel complex with the formula [Me_2_(DMAP)SiNIPr][OTf] was prepared by coordination with 4-dimethylamino-pyridine (DMAP). In the solid state, the DMAP adduct [Me_2_(DMAP)SiNIPr][OTf] contains a distinct [Me_2_(DMAP)SiNIPr]^+^ moiety.

## 1. Introduction

**Figure 1:**
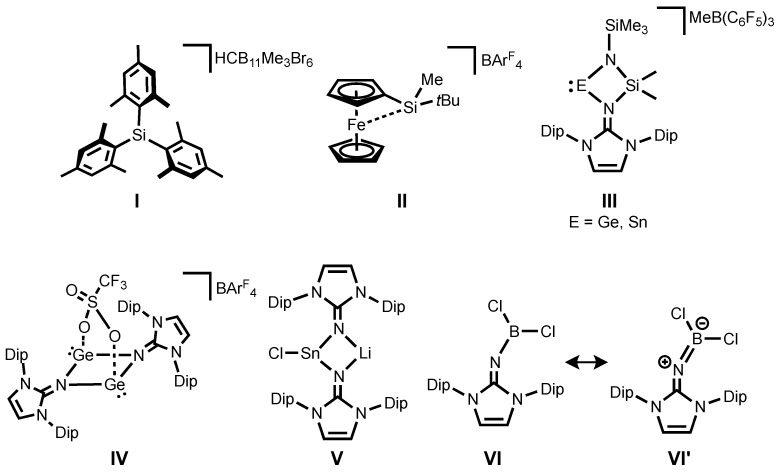
Selected group silylium ions **I** and **II** and metallyliumylidenes **III** and **IV** as well as bis(imino)stannylenoid **V**. Dichloroiminoborane **VI** and its canonical structure **VI’**. Dip = 2,6-diisopropylphenyl. Ar^F^ = 3,5-bis(trifluoromethyl)phenyl.

For decades, silicon analogues of carbenium ions, mostly referred to as silylium ions, have been attractive synthetic targets both for fundamental and applied research due to their electronic properties with strong Lewis acidity [1]. Since the pioneering work on silylium ions via hydride abstraction utilizing trityl cation by Corey and co-workers [2], several silylium ions were reported, although a weak interaction exists between a pronounced electrophilic silicon center and either solvent molecules or counteranions [3,4,5,6]. It should be emphasized that the first structurally authenticated free silylium ion **I** (Figure 1) was reported by Lambert, Reed and co-workers in 2002 [7]. Since then, several further examples of silyl cations have been reported [8,9,10]. On the other hand, the utilization of silylium ions as catalysts has also been an area of intense research interest and their catalytic activity has been examined in various reactions [11,12]. For example, the groups of Ozerov as well as Müller have independently reported the defluorination of fluoro- and perfluoro-alkyl groups by using silylium ions [13,14,15]. Moreover, Sawamura implemented a silylium ion as Lewis acid catalyst in Mukaiyama aldol and Diels-Alder reactions [16]. Catalytic C–C bond forming reactions have also been reported [17,18]. Recently, Oestreich and co-workers demonstrated that a ferrocene-based silylium ion **II** (Figure 1) displays catalytic activity in Diels-Alder reactions [19]. To date, most isolable silylium ions are typically limited to alkyl and aryl substituents and heteroatom-substituted silylium derivatives have remained extremely scarce. However, introduction of a heteroatom such as N, O, or S to the silylium center [20,21,22] produced silylium ions with good catalytic activity [23].

Tamm and co-workers developed a facile synthetic method for imidazolin-2-imino ligands and reported a number of metal complexes with these ligands [24,25,26,27]. During the last years, *N*-heterocyclic imines (NHIs) have been exploited for the syntheses of various unique main group compounds [28]. In Group 14, several metallylenes were synthesized by using NHIs as ligand [29,30,31,32], and two-coordinated metallyliumylidenes **III** (Figure 1) were also successfully isolated owing to stabilization of the NHI ligand by its high σ-donation together with delocalization of a positive charge around the imino fragment [31,32]. Shortly after, it was also demonstrated the NHIs effectively stabilize a spacer-separated bis(germyliumylidene) **IV** (Figure 1) [33]. Interestingly, their strong coordination ability enables the synthesis and isolation of a rare halogen-substituted stannylenoid **V** (Figure 1) [34] by coordinating of the imino-nitrogen to a Li cation, preventing the liberation of LiCl to form divalent tin species. Very recently, several imino-substituted borane compounds, of which the dichloro derivative **VI** (Figure 1) has a significant allenic-type character **VI’** (Figure 1) were reported [35]. Note that these iminoboranes act as Frustrated Lewis Pairs (FLPs), showing dehydrogenation of amine-borane. Motivated by this report and from the view point of their isoelectronic nature to silylium ions, iminosilylium ions would be an interesting synthetic target.

**Scheme 1:**
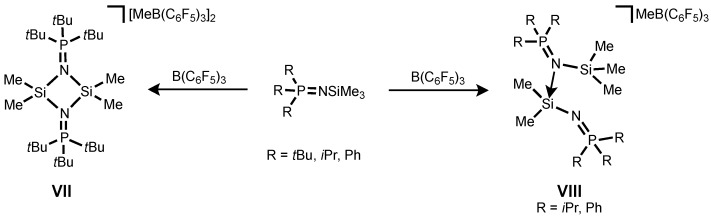
Syntheses of silylium ions **VII** and **VIII** via a methyl abstraction with B(C_6_F_5_)_3_.

It is noteworthy that the syntheses of phosphinoimido complexes of silylium ions were described by Stephan and co-workers and they exist as a dimer **VII** or a phosphinoimido-coordinated monomer **VIII** depending on the steric crowding of the substituents (*t*Bu vs. *i*Pr and Ph) (Scheme 1) [36].

Herein we describe the facile synthesis of novel imino-substituted silylium ions by the abstraction of methyl group of the trimethylsilyl ligand. Moreover, base-coordinated silylium ion was also prepared by treatment of Me_2_(OTf)SiNIPr with DMAP by expelling the OTf anion.

## 2. Results and Discussion

**Scheme 2:**
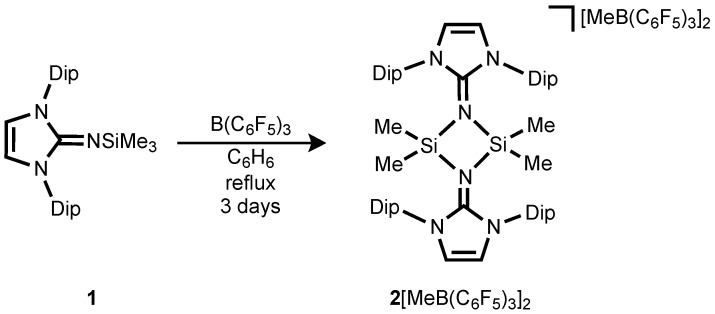
Synthesis of the dimeric silylium ion **2**[MeB(C_6_F_5_)_3_]. Dip = 2,6-diisopropylphenyl.

Reaction of Me_3_SiNIPr (**1**, NIPr = bis(2,6-diisopropylphenyl)imidazolin-2-imino) with B(C_6_F_5_)_3_ in benzene results in the formation of a biphasic mixture, in which the dark-red lower layer was found to contain the silylium ion **2**[MeB(C_6_F_5_)_3_]_2_ (Scheme 2). A signal was observed in the ^29^Si-NMR spectrum at δ = 17.3 ppm, which is significant low-field shifted compared to that of the precursor **1** (−22.3) [37]. This value is very similar to that of phosphinoimino complex of the silylium ion (δ(^29^Si) = 7.9 ppm) [36], illustrating the existence of dimer **2**[MeB(C_6_F_5_)_3_]_2_ in solution. The calculated chemical shift of optimized structure of **2** (8.1 ppm) corresponds well with the experimental value, supporting the presence of dimeric structure with a tetracoordinate silicon environment in **2**[MeB(C_6_F_5_)_3_]_2_. The ^19^F-NMR spectrum displays three distinct signals characteristic of [MeB(C_6_F_5_)_3_] due to the *ortho*, *meta*, and *para* F atoms of the C_6_F_5_ groups, which appear at similar shifts to those observed in **III** that have [MeB(C_6_F_5_)_3_] as a counterion [31,32]. Unfortunately, repeated efforts to grow single crystals of **2**[MeB(C_6_F_5_)_3_]_2_ suitable for X-ray diffraction analysis were all unsuccessful.

**Scheme 3:**
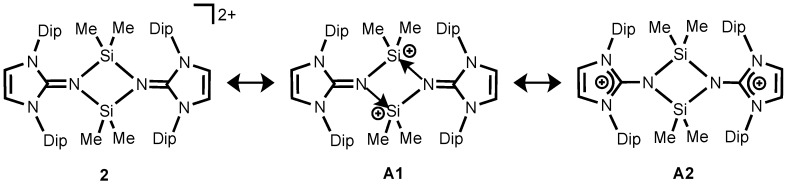
Selected resonance structures of **2** (Dip = 2,6-diisopropylphenyl).

**Figure 2:**
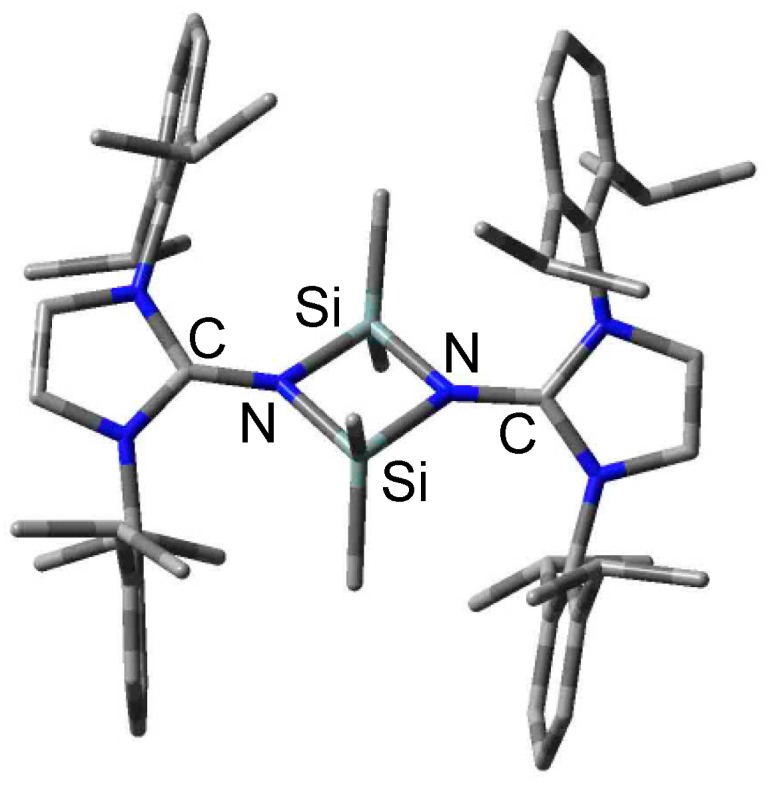
Calculated structure of **2**.

In order to gain deeper insight into the electronic nature of the structure of **2**, we performed a Natural Resonance Theory (NRT) analysis. Evaluation of the relative contributions of all important canonical forms for compound **2** shows that the imino-coordinated silylium ion formulation **A1** (78.9%) is dominant (Scheme 3). Additionally, compound **2** possesses significant imidazolium cation character **A2** (21.1%), where the formal positive charges reside on the both imidazoline rings. Consequently, the optimized structure of **2** has relatively long C–N_imino_ bonds (1.321 Å) which are comparable to those found in bis(germyliumylidene) **IV** (1.331(3), 1.335(2) Å) [33]. The calculated Si–N_imino_ bond length (1.820 Å) is significantly longer than that in the starting material **1** (1.677(2) Å) [26], indicating the high dative bond interaction between the silicon and the imino-nitrogen (Figure 2). The computed HOMO for **2** shows major π contributions located on the Dip unit (Figure S2). The LUMO shows N–C and C–C antibonding interactions on the imidazolium ring (Figure S2).

**Scheme 4:**
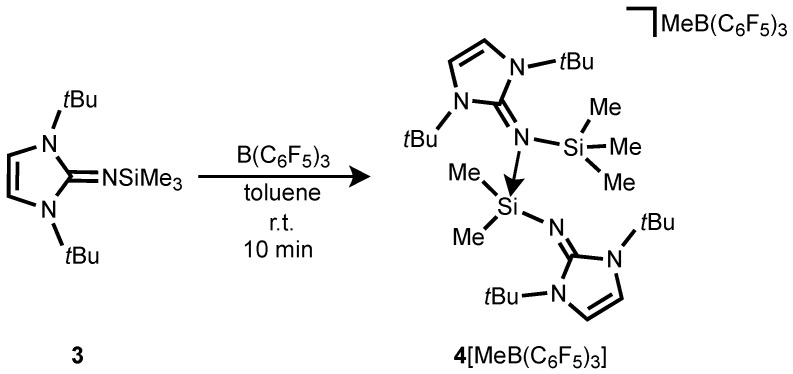
Synthesis of the Me_3_SiNI*t*Bu coordinated silylium ion **4**[MeB(C_6_F_5_)_3_].

The treatment of Me_3_SiNI*t*Bu **3** (NI*t*Bu = bis(*tert*-butyl)imidazolin-2-imino) with B(C_6_F_5_) in a 2:1 to form a two-layer reaction mixture (Scheme 4), accompanied by a color change from colorless to dark-red akin to **2**[MeB(C_6_F_5_)_3_]_2_. Although our efforts to obtain X-ray-quality crystals of this species have been unsuccessful, the NMR data as well as the ESI mass spectroscopy results are all consistent with the formulation of silylium salt [NI*t*BuSiMe_2_(NI*t*BuSiMe_3_)][MeB(C_6_F_5_)_3_] (**4**[MeB(C_6_F_5_)_3_]) (Scheme 4). This salt is formed by abstraction of a Si-bound methyl group, affording a silylium cation which is stabilized by intermolecular coordination of a second Me_3_SiNI*t*Bu molecule. The ^1^H-NMR spectrum shows two sets of signal for the imidazoline ligands. The two signals at δ = −0.01 and 0.34 in an integral ratio of 2:3 can be attributed to the Si-bound methyl groups. The ^29^Si-NMR spectrum reveals two resonances for **4**[MeB(C_6_F_5_)_3_] (δ(^29^Si) = −32.0, 13.1 ppm), in which the latter is assigned to the silicon atom of the silylium ion because this value is comparable to that of **2**[MeB(C_6_F_5_)_3_]_2_. The signals at −32.0 and 13.1 ppm are well reproduced by gauge-independent atomic orbitals (GIAO) calculation of the optimized structure of **4** and confirmed the assignment (−33.1 and 12.6 ppm). We also employed DFT calculations to assess the likely structure of the complex. The Me_2_Si–N_imino_ distance of 1.686 Å is comparable to that in **3** 1.655(2) Å) [24], meaning the covalent bond character. Alternatively, the contact between the coordinating imino ligand and the silylium center of 1.855 Å is significantly longer owing to the high dative bond character. The extent of the positive charge delocalization can be evaluated by the bond length of C_NHC_–N bond in the imino moiety. In this context, the bond distance of C_NHC_–N (1.374 Å) in the coordinating imine Me_3_SiNI*t*Bu is relatively longer than corresponding value of the main framework of [Me_2_SiNI*t*Bu]^+^ (1.313 Å), indicating the positive charge density is mainly delocalized into the coordinating silylimine ligand. Moreover, the bond angle of C_NHC_–N–Si (139.63°) shows a remarkably bent structure. This value is much larger than that in the iminosilylene-borane adduct having a significant silaimine character [29], indicating the π-electron interaction between the imino-nitrogen towards the silylium center can be neglected.

**Scheme 5:**
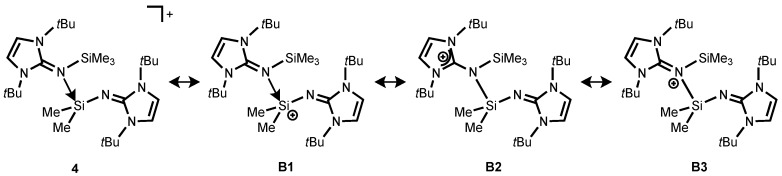
Selected resonance structures of **4**.

Moreover, the bonding situation of **4** were analyzed by means of NRT analysis (Scheme 5). The study shows that the dominant resonance form is **B1** (56.6%), in which the iminosilane (Me_3_SiNI*t*Bu) coordinates the vacant orbital on the silicon atom in the silylium ion (Scheme 5). As expected for the calculated structure of **4**, the resonance structure **B2** also has considerable weight (35.8%), with a positive charge being located on one of the imidazoline rings. In addition, compound **4** possesses a small contribution of the iminium ion **B3** (8.1%) (Scheme 5). Akin to **2**, HOMO is localized on imidazolin-2-imine fragment while LUMO is mainly on the π-contribution of the coordinated imidazolium ring (Figure S3).

When an excess amount of B(C_6_F_5_)_3_ is utilized, dimeric silylium [Me_2_SiNI*t*Bu]_2_[MeB(C_6_F_5_)_3_]_2_ is not formed. This different reactivity of Me_3_SiNIPr **1** and Me_3_SiNI*t*Bu **3** towards B(C_6_F_5_)_3_ can be caused by the steric bulk of the imidazoline fragment. This trend of the reactivity with B(C_6_F_5_)_3_ is very similar to phosphinoiminosilanes, where the bulky Me_3_SiNP(*t*Bu)_3_ formed the dimeric silylium ion **VII** while the less bulky silanes (Me_3_SiNP(R)_3_, R = *i*Pr, Ph) led to the formations of phosphinoimine-coordinated silylium ions **VIII** (Scheme 1) [36].

**Figure 3:**
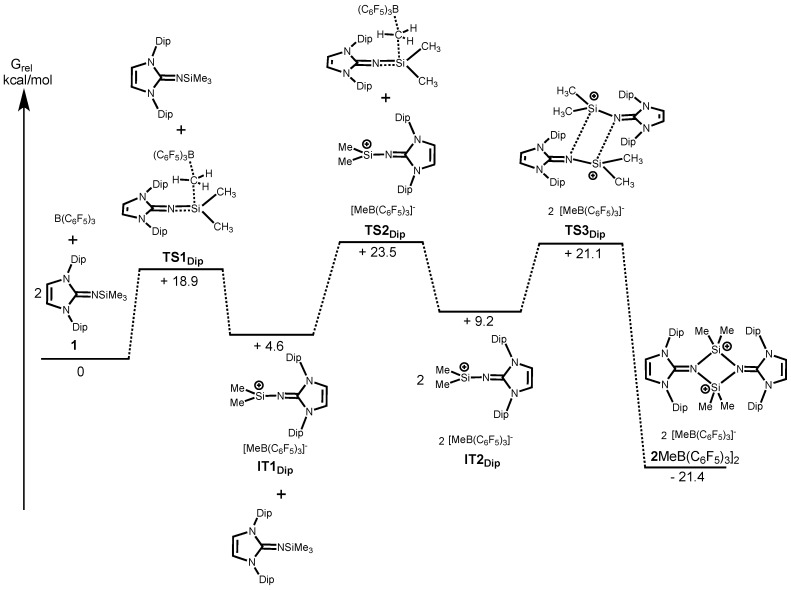
Reaction profile for the reaction of **1** with B(C_6_F_5_)_3_ (Dip = 2,6-diisopropylphenyl).

**Figure 4:**
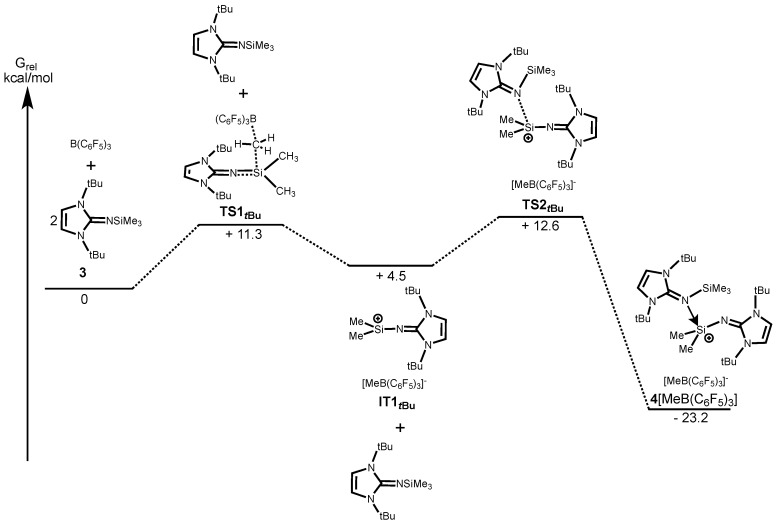
Reaction profile for the reaction of **3** with B(C_6_F_5_)_3_.

We next carried out the DFT calculations for elucidation of the mechanisms of formation of silylium ions **2**[MeB(C_6_F_5_)_3_]_2_ and **4**[MeB(C_6_F_5_)_3_], respectively. The difference in the steric bulk of the precursors **1** and **3** leads to different products based on the reaction kinetics. Figure 3 shows the energy profile along the reaction path calculated at the B97-D/6-31G(d) level of theory which has been successfully applied in low-valent silicon compounds [38,39,40,41,42,43,44,45,46,47]. The first step in the reaction is the abstraction of a Me group of a Me_3_Si moiety by the B(C_6_F_5_)_3_ (**TS1_Dip_**) with the associated energy of +18.9 kcal/mol for **2**[MeB(C_6_F_5_)_3_]_2_. On the other hand, the calculation revealed that a methyl-abstraction of **3** requires less energy (**TS1*_t_*_Bu_**, +11.3 kcal/mol) than that of **1** mainly due to the less sterically congested environment around the Si center of **3** (Figure 4). This finding is consistent with the experimental results, in which the reaction of **1** with B(C_6_F_5_)_3_ only proceeds at elevated temperature with prolonged reaction time, whereas **3** reacts with B(C_6_F_5_)_3_ instantly at room temperature. After abstracting of the first methyl group, a methyl-abstraction from the second Me_3_SiNIPr (**TS2_Dipp_**) with associated energy of +23.5 kcal/mol followed by the dimerization (**TS3_Dip_**) to form dicationic **2**[MeB(C_6_F_5_)_3_]_2_. Alternatively, coordination of the second Me_3_SiNI*t*Bu (**TS2*_t_*_Bu_**) with the associated energy of +12.6 kcal/mol leads to the imine-adduct **4**[MeB(C_6_F_5_)_3_]. We also calculated the reaction pathway with a concerted reaction that a precursor molecule supports a Me-group abstraction with B(C_6_F_5_)_3_ via its coordination to the back side of the forming silylium ion. However, it turned out that the corresponding transition state was not found. This is probably because the severe steric hindrance makes coordination of the second imine Me_3_SiNI*t*Bu to the silicon center highly unfavorable (Scheme S1).

To clarify the difference in the reactivity between Me_3_SiNIPr **1** and Me_3_SiNI*t*Bu **3**, the mechanism of the formation of [NIPrSiMe_2_(NIPrSiMe_3_)][MeB(C_6_F_5_)_3_] (**5**[MeB(C_6_F_5_)_3_]) and [Me_2_SiN*t*Bu]_2_[MeB(C_6_F_5_)_3_]_2_ (**6**[MeB(C_6_F_5_)_3_]_2_) were also carried out (see Supporting Materials). The associated energy of the coordination of the second Me_3_SiNIPr is relatively high (**TS2_Dip’_**, +26.7 kcal/mol) and the net energy gain of the formation **5**[MeB(C_6_F_5_)_3_] is small (−15.8 kcal/mol) mainly due to the steric repulsion between the bulky Dip groups (Figure S7). On the other hand, the net energy gain of the formation of the dication **6**[MeB(C_6_F_5_)_3_]_2_ (−18.9 kcal/mol) is smaller than that of **4**[MeB(C_6_F_5_)_3_] (−23.2 kcal/mol) (Figure S8).

We also considered the possibility of the direct Me abstraction from **4**[MeB(C_6_F_5_)_3_] to lead to **6**[MeB(C_6_F_5_)_3_]_2_. However, the transition state in this reaction is relatively high (25.2 kcal/mol) and it is an uphill process (+5.3 kcal/mol). Thus, this pathway to dimeric silylium ion **6**[MeB(C_6_F_5_)_3_]_2_ is both kinetic and thermodynamic unfavorable (Figure S9). Consequently, these theoretical results are also in accordance with the experimental results.

**Scheme 6:**
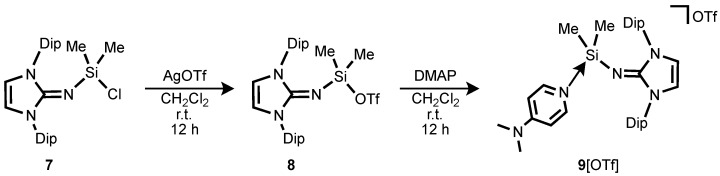
Synthesis of Me_2_Si(OTf)NIPr **8** and its DMAP adduct **9**[OTf] (Dip = 2,6-diisopropylphenyl, DMAP = 4-dimethylaminopyridine).

**Figure 5:**
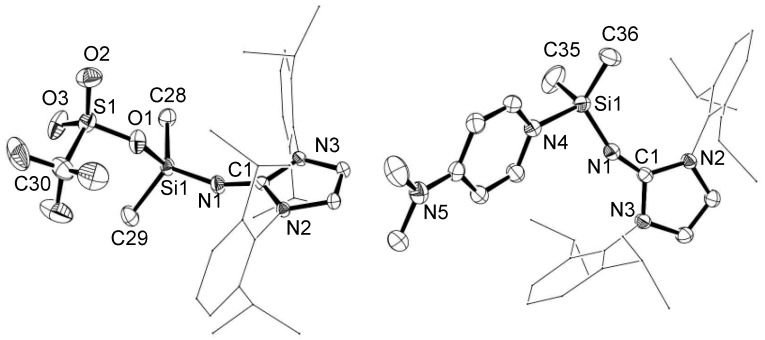
ORTEP representation of the molecular structures of **8** (**left**) and **9** (**right**) in the solid state. Thermal ellipsoids are at the 40% probability level. Hydrogen atoms are omitted for clarity. Dip groups are depicted as stick models. Selected bond lengths [Å] and bond angles [˚] for **8**: Si(1)−O(1) 1.7697(17), Si(1)−N(1) 1.6230(18), N(1)−C(1) 1.276(3), C(1)–N(2) 1.374(2), C(1)–N(3) 1.381(2), N(1)–Si(1) –O(1) 101.65(9), N(1)–Si(1)–C(29) 112.77(11), O(1)–Si(1)–C(29) 105.36(11), N(1)–Si(1)–C(28) 119.95(10), O(1)–Si(1)–C(28) 103.47(10), C(29)–Si(1)–C(28) 111.52(11), C(1)–N(1)–Si(1) 158.75(17). **9**: Si(1)−N(4) 1.8470(18), Si(1)−N(1) 1.6350(19), N(1)−C(1) 1.275(3); C(1)–N(2) 1.381(2), C(1)–N(3) 1.375(3), N(1)–Si(1)–N(4) 101.74(9), N(1)–Si(1)–C(36) 118.43(13), C(36)–Si(1)–N(4) 104.36(12), N(1)–Si(1)–C(35) 112.78(14), C(36)–Si(1)–C(35) 111.73(18), N(4)–Si(1)–C(35) 106.10(12), C(1)–N(1)–Si(1) 155.86(17).

To further explore the scope of iminosilylium ions, we have investigated different synthetic approaches. Treatment of Me_3_SiNIPr with one equivalent of Me_2_SiCl_2_ affords the Me_2_(Cl)SiNIPr **7** in high yield. The reaction of Me_2_(Cl)SiNIPr **7** with one equivalent of AgOTf results in the formation of iminotriflatosilane Me_2_(OTf)SiNIPr **8** by substitution of the chloride with a triflate group (Scheme 6). The observed ^29^Si resonance value for **8** (−18.5 ppm) was comparable with that of Me_2_Si(Cl)NIPr **7** (−19.2 ppm). Single crystal X-ray diffraction analysis of **8** reveals that it crystallizes as a contact ion pair with the triflate anion in the solid state to form a tetrahedral silicon center with an Si–O bond distance of 1.7696(17) Å (Figure 5). The bond angle of Si–N–C is 158.77(17)°, which falls in the range of the mono-imino-substituted tetravalent silanes [27,29]. Furthermore, the Si–N_imino_ bond distance (1.6231(18) Å) is comparable to those reported for tetravalent silane bearing an imino ligand and indicative of a negligible Si=N double bond character.

The reaction of **8** in toluene at 25 °C with one equivalent of DMAP affords a Lewis base adduct **9**[OTf] (Scheme 6). This complexation leads to displacement of the TfO–Si interaction in **8**. The ^29^Si-NMR spectrum of **9**[OTf] (in CD_2_Cl_2_) displays a singlet resonance at −16.4 ppm, which is slightly downfield shifted compared to those in **7** (−19.2 ppm) and **8** (−18.5 ppm). Compound **9**[OTf] crystallizes in monoclinic space group *P*2_1_/*c* as separated ion pairs (closest Si–anion separation: 4.699 Å). The molecular structure of **9**[OTf] is depicted in Figure 5. The Si–N_imino_ distance (1.6350(19) Å) is significantly shorter than the Si–N_DMAP_ separation (1.8470(18) Å), but is very close to the Si–N_imino_ bond length in **6** (1.6231(18) Å). Also, the N–C_NHC_ bond distance of 1.275(3) Å is a typical value for carbon-nitrogen double bonds in neutral imidazolin-2-imino compounds [26,27], indicating the delocalization of positive charge density into the imidazoline ring is negligible. Accordingly, instead of observing a linear Si–N–C angle in **9**, the Si–N–C angle is significantly bent (155.86(17)°). These structural features suggest little electron donation from the imino nitrogen to the silicon core, exhibiting no allenic-type bonding character.

**Scheme 7:**
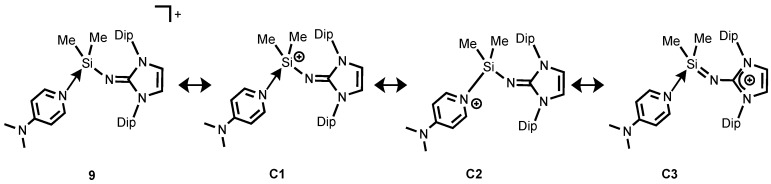
Selected resonance structures of **9**.

Judging from our NRT analysis, the relative contributions of resonance structures for compound **9** denotes that the DMAP-coordinated silylium ion **C1** is dominant (51.2%) (Scheme 7). Furthermore, **9** possesses significant pyridinium character represented by **C2**. Interestingly, the resonance form **C3** bearing the Si–N_imino_ double bond character, with formal positive charge being located at the imidazolium ring, has a non-negligible role in the description of **9** (18.5%, Scheme 7) despite the long Si–N_imino_ bond and the short N_imino_–C distance that observed in the X-ray structure.

## 3. Experimental Section

### 3.1. General Information

All experiments and manipulations were conducted under dry oxygen-free nitrogen using standard Schlenk techniques or in an MBraun glovebox workstation containing an atmosphere of purified nitrogen. Solvents were dried by standard methods. NMR solvents were degassed by multiple freeze-pump-thaw cycles and stored over molecular sieves (3 Å). Reagents were purchased from commercial suppliers and processed as received if not stated otherwise. The starting material **1** and **3** was prepared according to the reported procedure [25,26]. ^1^H-, ^11^B-, ^13^C{^1^H}-, and ^19^F-spectra were recorded on an Avance II 200 MHz or 400 MHz spectrometer (Bruker) and referenced to residual solvent signals as internal standards (^1^H and ^13^C) or an external standard (Et_2_O·BF_3_ for ^11^B, and CFCl_3_ for ^19^F). Values for the chemical shift (δ) are given in parts per million. Abbreviations: s = singlet; d = doublet; t = triplet; sept = septet. Elemental analyses and ESI-HRMS were carried out by the microanalytical laboratory and the MS-Service of the Institut für Chemie, Technische Universität Berlin, Germany, respectively.

### 3.2. Synthesis of **2**[MeB(C_6_F_5_)_3_]_2_

A Schlenk tube equipped with a PTFE-coated magnetic stirring bar was charged with Me_3_SiNIPr **1** (157 mg, 0.330 mmol) and B(C_6_F_5_)_3_ (175 mg, 0.342 mmol). Benzene (5 mL) was transferred to the reaction vessel by cannula and the resulting mixture was stirred under reflux condition for 72 h, resulting in a biphasic reaction mixture. The upper phase was removed, and the lower oily substance was washed with benzene. The oily residue was subjected to prolonged drying in high vacuum (6 h) to afford **2**[MeB(C_6_F_5_)_3_]_2_ as a pale red oil (144 mg, 44%); ^1^H-NMR (400 MHz, CD_2_Cl_2_): δ −0.19 (s, superimposed, 6H, Si(*CH_3_*)_2_), −0.19 (s, superimposed, 3H *Me*B(C_6_F_5_)_3_), 1.23 (d, ^3^*J*_HH_ = 7 Hz, 12H, CH(*CH_3_*)_2_), 1.35 (d, ^3^*J*_HH_ = 7 Hz, 12H, CH(*CH_3_*)_2_), 2.56 (sept, ^3^*J*_HH_ = 7 Hz, 4H, *CH*(CH_3_)_2_), 7.01 (s, 2H, N*CH*), 7.47 (d, ^3^*J*_HH_ = 8 Hz, 4H, Ar-H), 7.67 (t, ^3^*J*_HH_ = 8 Hz, 2H, Ar-H); ^11^B-NMR (64.2 MHz, CD_2_Cl_2_): δ = −16.6. ^13^C{^1^H}-NMR (100.6 MHz, CD_2_Cl_2_): δ = 0.3 (Si(CH_3_)_2_), 23.1 (CH(CH_3_)_2_), 25.3 (CH(CH_3_)_2_), 29.7 (CH(CH_3_)_2_), 120.2 (NCH), 126.4 (Ar-C), 128.5 (Ar-C), 133.6 (Ar-C), 146.9 (Ar-C), 148.0 (NCN). ^19^F-NMR (188.3 MHz, CD_2_Cl_2_): δ = −133.0 (s, 6F), −163.7 (t, *J* = 20 Hz, 3F), −167.6 (t, *J* = 20 Hz, 6F). ^29^Si{^1^H}-NMR (79.5 MHz, CD_2_Cl_2_): δ = 17.3.

### 3.3. Synthesis of **4**[MeB(C_6_F_5_)_3_]

A Schlenk tube equipped with a PTFE-coated magnetic stirring bar was charged with Me_3_SiNI*t*Bu 2 (355 mg, 1.33 mmol) and B(C_6_F_5_)_3_ (340 mg, 0.67 mmol). Toluene (10 mL) was transferred to the reaction vessel by cannula and the resulting mixture was stirred at room temperature for 1 h, resulting in a biphasic reaction mixture. The upper phase was removed, and the lower oily substance was washed with toluene. The oily residue was subjected to prolonged drying in high vacuum (6 h) to afford **4**[MeB(C_6_F_5_)_3_] as a pale red oil (672 mg, 48%); ^1^H-NMR (200 MHz, C_6_D_6_): δ −0.01 (s, 9H, Si(*CH_3_*)_3_), 0.34 (s, 6H, Si(*CH_3_*)_2_), 1.00 (s, 18H, N(*CH_3_*)_3_), 1.20 (s, 18H, N(*CH_3_*)_3_), 5.96 (s, 2H, N*CH*), 6.34 (s, 2H, N*CH*); ^11^B-NMR (64.2 MHz, C_6_D_6_): δ = −14.3; ^13^C{^1^H}-NMR (50.3 MHz, C_6_D_6_): δ = 4.3 (Si(CH_3_)_3_), 8.8 (Si(CH_3_)_2_), 28.7 (NC(CH_3_)_3_), 31.1 (NC(CH_3_)_3_), 55.2 (NC(CH_3_)_3_), 64.0 (NC(CH_3_)_3_), 108.6 (NCH), 117.7 (NCH), 141.9 (NCN), 147.0 (NCH). ^19^F-NMR (188.3 MHz, C_6_D_6_): δ = −131.8 (d, *J* = 20 Hz, 6F), −164.6 (t, *J* = 20 Hz, 3F), −167.0 (t, *J* = 20 Hz, 6F). ^29^Si{^1^H}-NMR (79.5 MHz, CD_2_Cl_2_): δ = 13.1, −32.0; ESI-HRMS: *m*/*z*: 519.4031 (calcd. 519.4021 for [M − MeB(C_6_F_5_)_3_]^+^).

### 3.4. Synthesis of Me_2_(Cl)SiNIPr *(**7**)*

To a stirring mixture of Me_3_SiNIPr (1.88 g, 3.95 mmol) in toluene (10 mL) was added Me_2_SiCl_2_ (0.65 mL, 5.39 mmol) via syringe at −78 °C. The reaction mixture was stirred for 12 h while allowed to slowly warm to room temperature. The solvent was removed under vacuum and washed with hexane to give Me_2_(Cl)SiNIPr as a yellow solid (1.55 g, 79%). Recrystallization from hexane (3 mL) afforded Me_2_(Cl)SiNIPr in the form of colorless crystals; ^1^H-NMR (200 MHz, C_6_D_6_): δ 0.10 (s, 6H, Si*Me_2_*), 1.16 (d, ^3^*J*_HH_ = 7 Hz, 12H, CH(*CH_3_*)_2_), 1.39 (d, ^3^*J*_HH_ = 7 Hz, 12H, CH(*CH_3_*)_2_), 3.07 (sept, ^3^*J*_HH_ = 7 Hz, 4H, *CH*(CH_3_)_2_), 5.94 (s, 2H, N*CH*), 7.10–7.26 ppm (m, 6 H, Ar-H); ^13^C{^1^H}-NMR (50.3 MHz, C_6_D_6_): δ 6.3 (Si(*C*H_3_)_2_), 23.5 (CH(*C*H_3_)_2_), 24.5 (CH(*C*H_3_)_2_), 29.0 (*C*H(CH_3_)_2_), 114.4 (N*C*H), 124.1 (Ar-C), 129.9 (Ar-C), 134.2 (Ar-C), 143.0 ppm (N*C*N), 147.7 (Ar-C); ^29^Si{^1^H}-NMR (79.5 MHz, C_6_D_6_): δ −19.2; Elemental analysis calcd. (%) for C_29_H_42_ClN_3_Si: C 70.20, H 8.53, N 8.47; found: C 69.69, H 8.33, N 9.10. m.p. at 155–158 °C (dec.).

### 3.5. Synthesis of Me_2_(OTf)SiNIPr *(**8**)*

A Schlenk tube equipped with a PTFE-coated magnetic stirring bar was charged with Me_2_ClSiNIPr **7** (133 mg, 0.268 mmol) and Ag[OTf] (69 mg, 0.268 mmol). CH_2_Cl_2_ (5 mL) was transferred to the reaction vessel by cannula at room temperature and the resulting mixture was stirred for 12 h. The volatiles were removed under reduced pressure and the residue was recrystallized from CH_2_Cl_2_ (5 mL) at −30 °C to afford Me_2_(OTf)SiNIPr (**8**) as colorless crystals (107 mg, 65%). The batch contained crystals suitable for single crystal X-ray diffraction analysis. ^1^H-NMR (200 MHz, C_6_D_6_): δ 0.00 (s, 6H, Si*Me_2_*), 1.10 (d, ^3^*J*_HH_ = 7 Hz, 12H, CH(*CH_3_*)_2_), 1.30 (d, ^3^*J*_HH_ = 7 Hz, 12H, CH(*CH_3_*)_2_), 2.91 (sept, ^3^*J*_HH_ = 7 Hz, 4H, *CH*(CH_3_)_2_), 5.89 (s, 2H, N*CH*), 7.07–7.25 ppm (m, 6 H, Ar-H); ^13^C{^1^H}-NMR (100.6 MHz, C_6_D_6_): δ 2.10 (Si(*C*H_3_)_2_), 23.3 (CH(*C*H_3_)_2_), 24.4 (CH(*C*H_3_)_2_), 29.0 (*C*H(CH_3_)_2_), 114.7 (N*C*H), 124.3 (Ar-C), 130.2 (Ar-C), 133.4 (Ar-C), 143.5 ppm (N*C*N), 147.3 (Ar-C); ^29^Si{^1^H}-NMR (79.5 MHz, C_6_D_6_): δ −18.5; ESI-HRMS: *m*/*z*: 462.3480 (calcd. 462.3299 for [M − OTf + 2 H]^+^). 208–210 °C (dec.).

### 3.6. Synthesis of [Me_2_(DMAP)SiNIPr][OTf] **9**[OTf]

A Schlenk tube equipped with a PTFE-coated magnetic stirring bar was charged with Me_2_(OTf)SiNIPr **8** (106 mg, 0.174 mmol) and DMAP (26 mg, 0.213 mmol). CH_2_Cl_2_ (5 mL) was transferred to the reaction vessel by cannula at room temperature and the resulting mixture was stirred for 12 h. The volatiles were removed under reduced pressure and the residue was recrystallized from CH_2_Cl_2_ (5 mL) at −30 °C to afford [Me_2_(DMAP)SiNIPr][OTf] (**9**[OTf]) as colorless crystals (53 mg, 42%). The batch contained crystals suitable for single crystal X-ray diffraction analysis. ^1^H-NMR (400.1 MHz, CD_3_CN): δ = −0.21 (s, 6H, Si(C*H*_3_)_2_), 1.17 (d, ^3^*J*_HH_ = 7 Hz, 12H, CH(C*H*_3_)_2_), 1.21 (d, ^3^*J*_HH_ = 7 Hz, 12H, CH(C*H*_3_)_2_), 2.87 (sept, ^3^*J*_HH_ = 7 Hz, 4 H, C*H*(CH_3_)_2_), 3.08 (s, 6H, N(C*H*_3_)_2_), 6.36 (d, ^3^*J*_HH_ = 7 Hz, 2 H, DMAP-H-2,6 or -3,5), 6.82 (s, 2 H, NC*H*), 7.31 ppm (d, ^3^*J*_HH_ = 7 Hz, 2H, DMAP-H-2,6 or -3,5), 7.40 (d, ^3^*J*_HH_ = 8 Hz, 4H, Ar-H), 7.56 (t, ^3^*J*_HH_ = 8 Hz, 2H, Ar-H); ^13^C{^1^H}-NMR (100.6 MHz, CD_2_Cl_2_): δ 1.6 (Si(*C*H_3_)_2_), 23.1 (CH(*C*H_3_)_2_), 24.7 (CH(*C*H_3_)_2_), 29.1 (*C*H(CH_3_)_2_), 40.1(N(CH_3_)_2_), 107.4 (DMAP-C-2,6 or -3,5), 115.5 (N*C*H), 124.7 (Ar-C), 130.6 (Ar-C), 133.3 (Ar-C), 142.8 (DMAP-C-2,6 or -3,5), 147.9 (Ar-C), 155.1 (DMAP-C-4), 156.8 ppm (N*C*N); ^19^F-NMR (188.3 MHz, CD_2_Cl_2_): δ = −78.9; ^29^Si{^1^H}-NMR (79.5 MHz, CD_2_Cl_2_): δ = −16.4 ppm; ESI-HRMS: *m*/*z*: 592.3985 (calcd. 592.3586 for [M − OTf]^+^). 173–176 °C (dec.).

### 3.7. X-ray Crystallography

X-ray data collection and structural refinement. Intensity data for compounds **7**, **8** and **9**[OTf] collected on an Agilent SuperNova diffractometer, equipped with a CCDC area Atlas detector and a mirror monochromator utilizing Cu*K*_α_ radiation (λ = 1.54184 Å). CCDC deposition numbers: 1491068 for **7**, and 1491070 for **8**, 1491069 for **9**[OTf]. The data can be obtained free of charge from the Cambridge Crystallography Data Center via http://www.ccdc.cam.ac.uk/conts/retrieving.html (or from the CCDC, 12 Union Road, Cambridge CB2 1EZ, UK; Fax: +44 1223 336033; E-mail: deposit@ccdc.cam.ac.uk).

## 4. Conclusions

In summary, we have synthesized imino-substituted silylium ions **2**[MeB(C_6_F_5_)_3_]_2_ and **4**[MeB(C_6_F_5_)_3_] by the straightforward Me-abstraction of trimethylsilylimines **1** and **2**, respectively. Depending on the steric demand of the imines, the reaction leads to an unusual dimeric silylium ion bridged by the NHI ligands and a Me_3_SiNI*t*Bu-coordinated silicon monocation. The mechanistic details of the formation of **2**[MeB(C_6_F_5_)_3_]_2_ and **4**[MeB(C_6_F_5_)_3_] was elucidated by theoretical calculations. Moreover, the iminotriflatosilane and its DMAP adduct were also prepared. The former exhibits the contact ion pair of the silylium and the triflate while **9**[OTf] has a dative bond between the Si atom and DMAP and no interaction with the triflate. Future investigations will be focused on the substitution of a triflate group to a weakly coordinating anion such as B(C_6_F_5_)_4_^−^ or Al[OC(CF_3_)_3_]_4_^−^ in order to isolate the three coordinate iminosilylium ion.

## Supplementary Materials

Supplementary materials can be accessed at: http://www.mdpi.com/1420-3049/21/9/1155/s1.
