# Supplementary material for: Facile Access to Stable Silylium Ions Stabilized by N-Heterocyclic Imines
Source: Molecules. 2016 Aug 30;21(9):1155. doi: 10.3390/molecules21091155 (PMC6273809; doi:10.3390/molecules21091155)
Supplement: Supplementary file 1 [file molecules-21-01155-s001.pdf]

Supplementary Materials: Facile Access to Stable Silylium Ions Stabilized by *N*-Heterocyclic Imines

Tatsumi Ochiai, Tibor Szilvási and Shigeyoshi Inoue

S1. Crystallographic Data for 7, 8, 9[OTf]

General Considerations: Data for the single crystal structure determinations of **7**, **8** and **9**[OTf] were collected on an Agilent SuperNova diffractometer, equipped with a CCDC area Atlas detector and a mirror monochromator utilizing CuK_α radiation (λ = 1.54184 Å). The crystal structures were solved by Direct Methods and refined on F^2 using full-matrix least squares with SHELXL-97 [1]. The positions of the H atoms at the carbon atoms were calculated by standard methods. CCDC deposition numbers: 1491068 for **7**, and 1491070 for **8**, 1491069 for **9**[OTf].

Table S1. Crystal data and structure refinement for **7**.

Empirical formula	$C_{29}H_{42}ClN_3Si$	
Formula weight	496.2	
Temperature	150(2) K	
Wavelength	1.54184 Å	
Crystal system	Triclinic	
Space group	*P*-1	
Unit cell dimensions	a = 9.5590(2) Å	α = 88.352(2)°
	b = 12.4732(4) Å	β = 83.833(2)°
	c = 25.2203(7) Å	γ = 78.766(2)°
Volume	2932.27(14) Å^3	
Z	4	
Density (calculated)	1.124 Mg/m^3	
Absorption coefficient	1.687 mm^{-1}	
F(000)	1072	
Crystal size	0.402 × 0.096 × 0.038 mm^3	
Theta range for data collection	3.53 to 67.50°	
Index ranges	−11<=h<=9, −14<=k<=14, −30<=l<=29	
Reflections collected	20006	
Independent reflections	10553 [R(int) = 0.0262]	
Completeness to theta = 67.50°	99.90%	
Absorption correction	Semi-empirical from equivalents	
Max. and min. transmission	1.00000 and 0.32135	
Refinement method	Full-matrix least-squares on F^2	
Data/restraints/parameters	10553/6/632	
Goodness-of-fit on F^2	1.052	
Final R indices [I > 2sigma(I)]	R1 = 0.0518, wR2 = 0.1454	
R indices (all data)	R1 = 0.0607, wR2 = 0.1551	
Largest diff. peak and hole	1.008 and −0.761 e.Å^{-3}	

Table S2. Crystal data and structure refinement for **8**.

Empirical formula	$C_{30}H_{42}F_3N_3O_3SSi$
Formula weight	609.82
Temperature	149.99(10) K
Wavelength	1.54180 Å
Crystal system	Monoclinic

Table S2. *Cont.*

Space group	$P2_1/c$	
Unit cell dimensions	a = 10.93550(10) Å	α = 90°
	b = 17.13290(10) Å	β = 99.0410(10)°
	c = 18.01170(10) Å	γ = 90°
Volume	3332.69(4) $Å^3$	
Z	4	
Density (calculated)	1.215 Mg/m^3	
Absorption coefficient	1.631 mm^{-1}	
F(000)	1296	
Crystal size	0.62 × 0.36 × 0.23 mm^3	
Theta range for data collection	3.58 to 67.50°	
Index ranges	−12<=h<=13, −20<=k<=20, −21<=l<=18	
Reflections collected	12722	
Independent reflections	5950 [R(int) = 0.0159]	
Completeness to theta = 67.50°	99.10%	
Absorption correction	Semi-empirical from equivalents	
Max. and min. transmission	1.00000 and 0.4312	
Refinement method	Full-matrix least-squares on F^2	
Data/restraints/parameters	5951/0/380	
Goodness-of-fit on F^2	1.031	
Final R indices [I > 2sigma(I)]	R1 = 0.0539, wR2 = 0.1376	
R indices (all data)	R1 = 0.0565, wR2 = 0.1396	
Largest diff. peak and hole	1.137 and −0.467 e.$Å^{-3}$	

Table S3. Crystal data and structure refinement for **9**[OTf].

Empirical formula	$C_{37}H_{52}F_3N_5O_3SSi$	
Formula weight	731.99	
Temperature	150.01(16) K	
Wavelength	1.54184 Å	
Crystal system	Monoclinic	
Space group	$P2_1/c$	
Unit cell dimensions	a = 10.3153(2) Å	α = 90°
	b = 17.1502(3) Å	β = 100.2030(10)°
	c = 22.8540(3) Å	γ = 90°
Volume	3979.15(12) $Å^3$	
Z	4	
Calculated density	1.222 Mg/m^3	
Absorption coefficient	1.465 mm^{-1}	
F(000)	1560	
Crystal size	0.27 × 0.11 × 0.10 mm^3	
Theta range for data collection	3.24 to 67.49°	
Limiting indices	−11<=h<=12, −20<=k<=18, −19<=l<=27	
Reflections collected/unique	15496/7162 [R(int) = 0.0283]	
Completeness to theta = 67.49	99.90%	
Absorption correction	Semi-empirical from equivalents	
Max. and min. transmission	1.00000 and 0.40955	
Refinement method	Full-matrix least-squares on F^2	
Data/restraints/parameters	7162/0/463	
Goodness-of-fit on F^2	1.023	
Final R indices [I > 2sigma(I)]	R1 = 0.0479, wR2 = 0.1264	
R indices (all data)	R1 = 0.0622, wR2 = 0.1393	
Largest diff. peak and hole	0.553 and −0.408 e.$Å^{-3}$	

S1.1. Molecular Structure of **7** *in the Solid State*

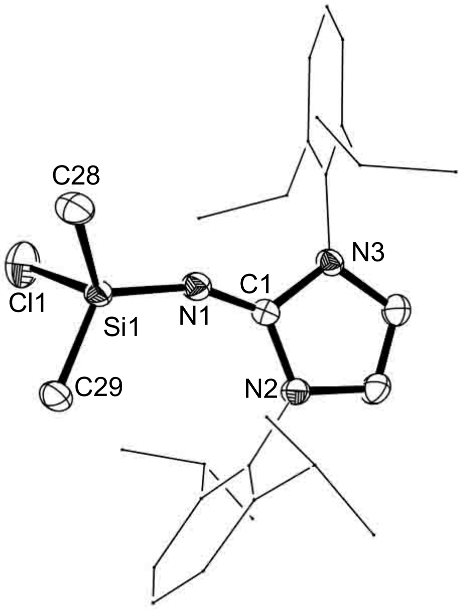

Figure S1. ORTEP representation of the molecular structure of one of the two independent molecules of **7** in the solid state. Thermal ellipsoids are at the 40% probability level. Hydrogen atoms are omitted for clarity. Dip groups are depicted as stick models. Selected bond lengths (Å) and bond angles (°); values for the not depicted molecules are given in square brackets: Si1–Cl1, 2.0975(9), [2.0766(9)]; Si1–N1, 1.6425(18), [1.6413(18)]; Si1–C29, 1.858(2), [1.857(3)]; Si1–C28, 1.861(2), [1.886(2)]; N1–C1, 1.273(3), [1.268(3)]; C1–N2, 1.383(3), [1.387(3)]; C1–N3, 1.387(3), [1.385(3)].

S2. Details to the DFT Calculations

DFT calculations for electronic structure analysis were performed at the B3LYP/6-31G* level of theory while energy differences were calculated at B97-D/6-31G* level [2–7]. GIAO NMR chemical shifts were calculated at B3LYP/6-311G** level. Stationary points on the potential energy surface (PES) were characterized by harmonic vibrational frequency calculations. We calculated the electronic structure analysis at B97-D/6-31G* level as well and found generally negligible difference compared to B3LYP/6-31G* supporting that our results are independent from the chosen method. All calculations were carried out using GAUSSIAN 09 program [8,9].

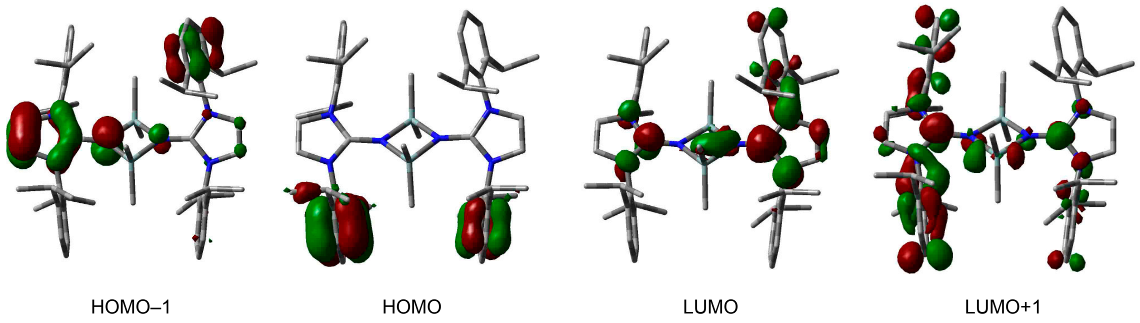

Figure S2. Kohn-Sham depictions of molecular orbitals (HOMO–1, HOMO, LUMO, LUMO+1) of **2**.

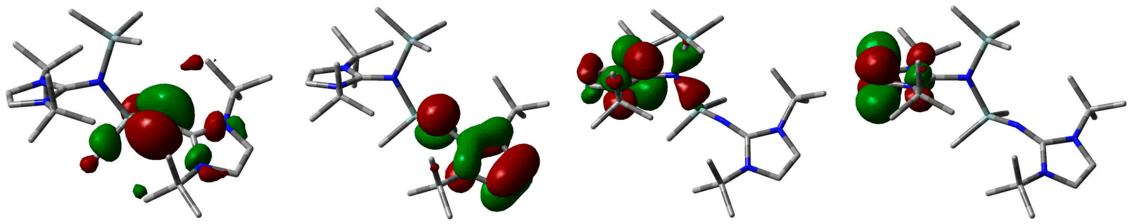

HOMO–1 HOMO LUMO LUMO+1

Figure S3. Kohn-Sham depictions of molecular orbitals (HOMO–1, HOMO, LUMO, LUMO+1) of **4**.

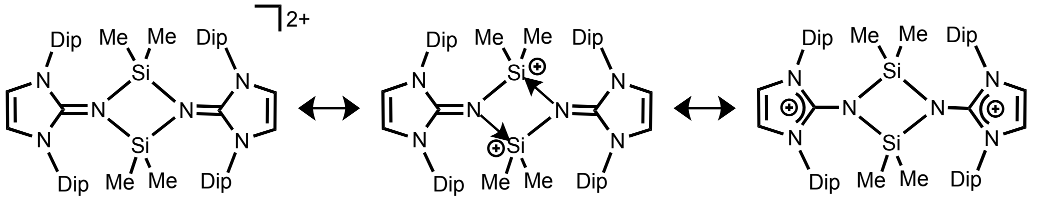

2 **A1** 78.9 % **A2** 21.1 %

Figure S4. NRT-analysis of **2**.

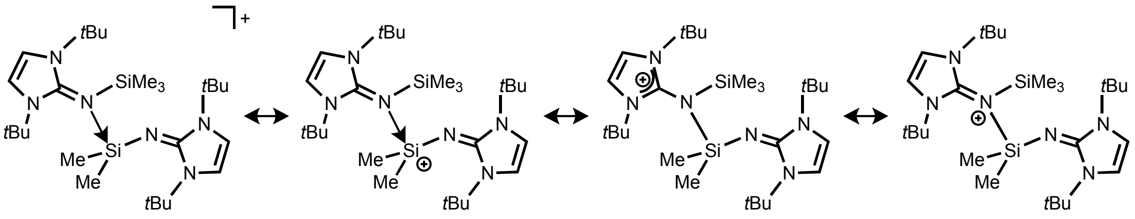

4 **B1** 56.1 % **B2** 35.8 % **B3** 8.1 %

Figure S5. NRT-analysis of **4**.

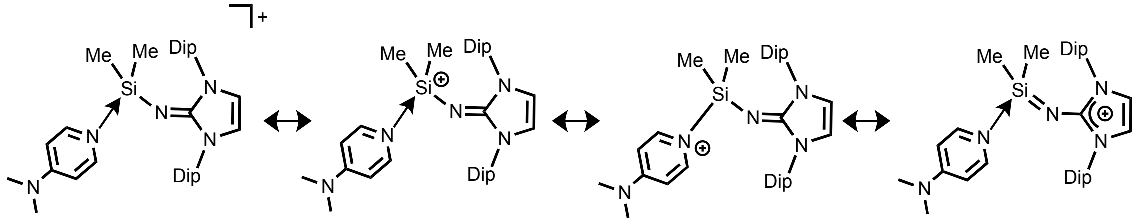

9 **C1** 51.2 % **C2** 30.3 % **C3** 18.5 %

Figure S6. NRT-analysis of **9**.

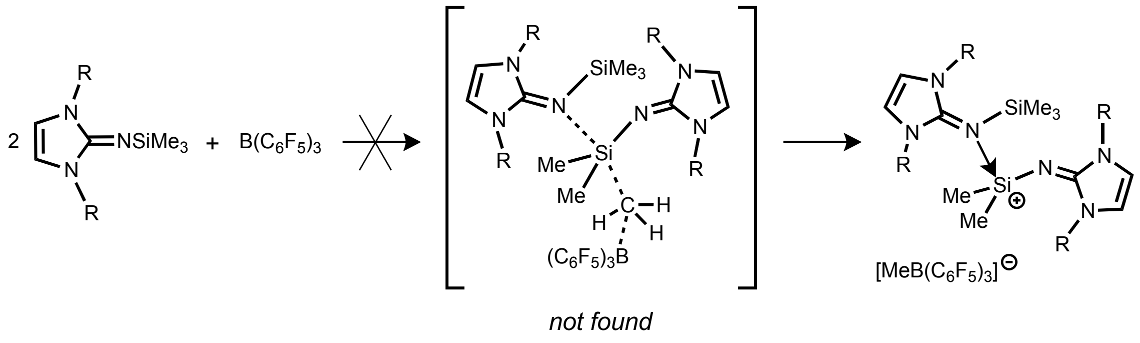

Scheme S1. Reaction pathway involving a methyl abstraction supported by a precursor molecule.

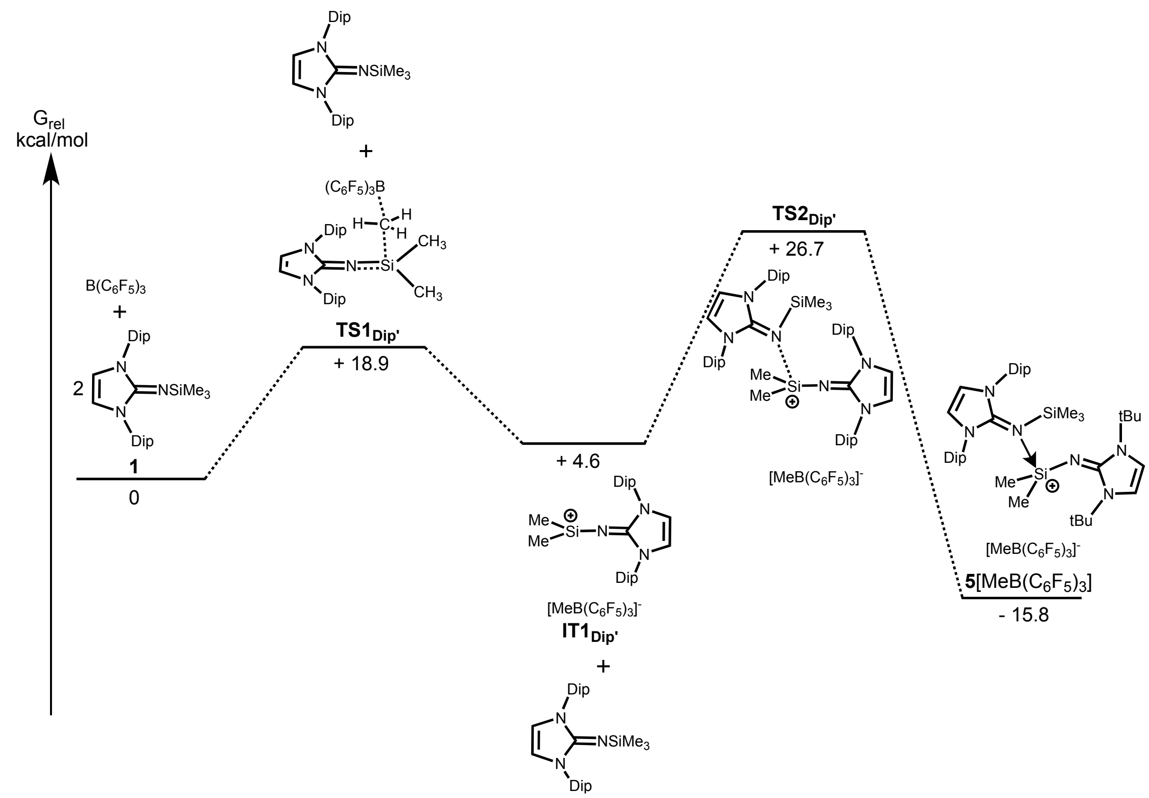

Figure S7. Reaction profile for the formation of the hypothetical silylium ion **5**[MeB(C_6F_5)$_3$] (Dip = 2,6-diisopropylphenyl).

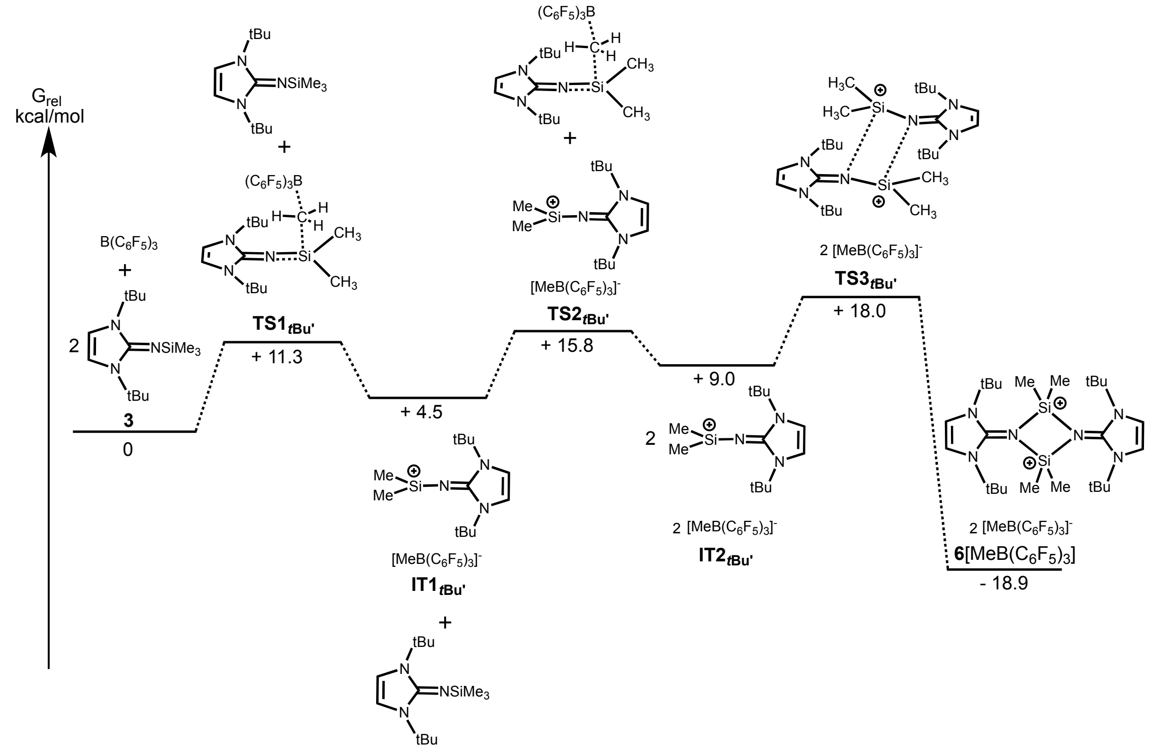

Figure S8. Potential energy profile for the formation of the hypothetical silylium ion **6**[MeB(C_6F_5)$_3$]$_2$.

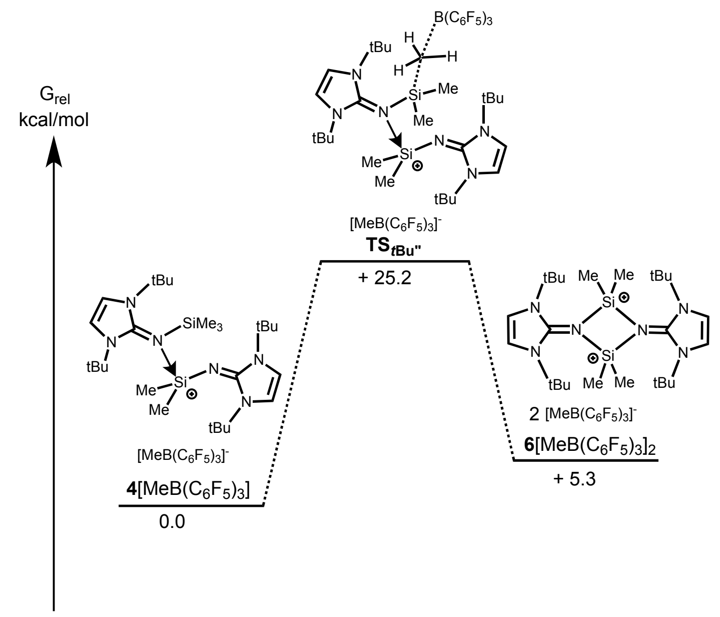

Figure S9. Potential energy profile for the formation of the hypothetical silylium ion **6**[MeB(C_6F_5)$_3$]$_2$.

Table S4. Cartesian geometry of B(C_6F_5)$_3$ in Angstrom [Å].

Atomtype	X Coordinates	Y Coordinates	Z Coordinates
C	2.34909	−0.893652	−0.717067
C	1.146691	−1.077347	−0.001193
C	1.04054	−2.288618	0.715519
C	2.053799	−3.253661	0.736068
C	3.227397	−3.027831	−0.000249
C	3.37774	−1.842197	−0.736519
B	−0.000925	−0.000292	−0.000801
C	−1.507107	−0.455628	−0.000361
C	−2.503242	0.244135	0.714023
C	−3.84602	−0.149202	0.733626
C	−4.237589	−1.279743	−0.000603
C	−3.285817	−2.004974	−0.734589
C	−1.949499	−1.589948	−0.714318
F	−2.17636	1.328123	1.449191
F	−4.758922	0.535965	1.441827
F	−5.519081	−1.66678	−0.000109
F	−3.667011	−3.081034	−1.442531
F	−1.077058	−2.31306	−1.448069
F	−0.060475	−2.547354	1.452577
F	1.919396	−4.385499	1.446972
F	4.20376	−3.94371	−0.000104
F	4.499361	−1.634398	−1.445827
F	2.536768	0.22201	−1.453657
C	0.358413	1.531755	0.000089
C	1.463303	2.04392	0.713791
C	1.794293	3.403426	0.734132
C	1.010837	4.308261	0.000875
C	−0.093351	3.847371	−0.733138

Table S4. *Cont.*

Atomtype	X Coordinates	Y Coordinates	Z Coordinates
C	−0.402358	2.482664	−0.713646
F	2.239536	1.218632	1.447642
F	−1.464452	2.089404	−1.448309
F	−0.83437	4.716291	−1.440401
F	1.316923	5.611417	0.001409
F	2.844418	3.851003	1.442206

Table S5. Cartesian geometry of **1** in Angstrom [Å].

Atomtype	X Coordinates	Y Coordinates	Z Coordinates
C	2.778565	−1.256836	−0.50686
C	2.315422	0.047797	−0.803515
C	3.168377	1.177172	−0.82163
C	4.532483	0.967657	−0.551562
C	5.020186	−0.314469	−0.271022
C	4.151603	−1.412877	−0.244322
N	0.935521	0.232175	−1.13235
C	−0.137591	0.177802	−0.218634
N	−1.268924	0.335306	−1.044993
C	−0.893731	0.465715	−2.384001
C	0.462093	0.413012	−2.439879
C	−2.608563	0.100603	−0.593924
C	−3.427215	1.207056	−0.271203
C	−4.733618	0.942481	0.175913
C	−5.199392	−0.37401	0.300398
C	−4.3666	−1.45062	−0.024876
C	−3.054247	−1.235117	−0.48341
N	−0.162479	0.017046	1.045137
Si	0.616553	−0.084767	2.565702
C	0.409004	−1.852449	3.259051
C	−2.873105	2.628485	−0.346083
C	−2.159887	2.974454	0.983822
C	−2.147377	−2.41903	−0.808304
C	−1.753901	−3.159044	0.489447
C	2.598612	2.574353	−1.079245
C	3.6443	3.592322	−1.575793
C	1.812916	−2.443251	−0.482735
C	2.317901	−3.632927	0.357704
C	−3.938382	3.690612	−0.687206
C	−2.79366	−3.383699	−1.826288
C	1.892797	3.111246	0.190268
C	1.480579	−2.918392	−1.917663
C	2.475498	0.344474	2.63424
H	−1.63797	0.580268	−3.163232
H	1.148982	0.484198	−3.274728
H	−5.392248	1.772705	0.433316
H	−6.216479	−0.559133	0.653682
H	−4.733458	−2.473859	0.077851
H	−2.113237	2.651727	−1.142967

Table S5. *Cont.*

Atomtype	X Coordinates	Y Coordinates	Z Coordinates
H	−4.494948	3.428161	−1.601385
H	−3.449976	4.666135	−0.840782
H	−4.662846	3.810895	0.134825
H	−2.891847	2.997588	1.808616
H	−1.68262	3.966761	0.914449
H	−1.398045	2.220268	1.224918
H	−1.222164	−2.03813	−1.261501
H	−3.708447	−3.84222	−1.416749
H	−2.089077	−4.195714	−2.070403
H	−3.060017	−2.859488	−2.758137
H	−1.264171	−2.464003	1.184092
H	−1.060242	−3.986106	0.261534
H	−2.645072	−3.583501	0.981501
H	5.220869	1.812968	−0.557585
H	6.082989	−0.457778	−0.063796
H	4.546228	−2.401572	−0.009628
H	1.829615	2.487595	−1.862604
H	1.407768	4.077498	−0.027407
H	2.629921	3.267931	0.994684
H	1.13119	2.408755	0.554981
H	4.377231	3.829746	−0.787307
H	3.140044	4.531279	−1.853241
H	4.192207	3.215188	−2.454175
H	0.879705	−2.097833	−0.016589
H	1.044141	−2.10897	−2.520391
H	0.75534	−3.748186	−1.879228
H	2.392766	−3.279047	−2.421737
H	3.176618	−4.13219	−0.121549
H	1.511703	−4.37654	0.455787
H	2.621185	−3.314013	1.36671
H	2.827328	0.277253	3.68023
H	3.093056	−0.333305	2.025653
H	2.672455	1.36971	2.284326
H	0.844324	−2.615227	2.593251
H	−0.654913	−2.104793	3.402419
H	0.909654	−1.938367	4.239904
C	−0.277111	1.113648	3.753208
H	0.10779	1.012766	4.783691
H	−0.140011	2.165084	3.447225
H	−1.361643	0.913541	3.771714

Table S6. Cartesian geometry of transition state (+18.9 kcal/mol) in Figure 3 in Angstrom [Å].

Atomtype	X Coordinates	Y Coordinates	Z Coordinates
C	4.352194	−2.955952	−0.732771
C	3.333584	−2.625185	0.19029
C	2.366677	−3.554531	0.635925
C	2.457015	−4.868286	0.141942
C	3.45072	−5.226261	−0.778112
C	4.382555	−4.276703	−1.216214
N	3.297161	−1.278814	0.69451

Table S6. *Cont.*

Atomtype	X Coordinates	Y Coordinates	Z Coordinates
C	2.865441	−0.194838	−0.053386
N	3.271545	0.92173	0.659097
C	3.932838	0.525874	1.820804
C	3.954829	−0.835157	1.842429
N	2.28607	−0.174115	−1.217698
Si	1.280743	−0.896456	−2.293771
C	1.109124	0.066112	−3.902007
C	3.03876	2.284553	0.245641
C	3.708671	2.756584	−0.912117
C	3.431092	4.07051	−1.324719
C	2.542504	4.883214	−0.609168
C	1.933339	4.403901	0.55217
C	2.165018	3.093075	1.00916
C	4.756556	1.903617	−1.632358
C	6.078883	1.896608	−0.827039
C	1.535232	2.631269	2.321199
C	0.026341	2.94398	2.409424
C	1.268233	−3.145977	1.610673
C	1.637339	−3.553584	3.056583
C	5.397498	−1.946319	−1.203451
C	5.251212	−1.655749	−2.713766
C	−1.196802	−0.76399	−1.492141
B	−2.333705	−0.075352	−0.440723
C	−2.037544	−0.569955	1.128766
C	−0.775271	−0.360657	1.697053
C	−0.422138	−0.681634	3.011096
C	−1.372082	−1.279746	3.841947
C	−2.665744	−1.485706	3.347391
C	−2.97464	−1.112297	2.02739
F	0.225334	0.194628	0.944708
F	0.841491	−0.482369	3.467327
F	−1.037455	−1.661204	5.091152
F	−3.60347	−2.025934	4.150414
F	−4.262878	−1.295315	1.667479
C	−2.249305	1.57981	−0.375884
C	−3.095663	2.270045	0.510954
C	−3.043522	3.651258	0.735036
C	−2.106492	4.419873	0.031669
C	−1.264993	3.793281	−0.890985
C	−1.349646	2.403076	−1.06543
F	−4.039007	1.582951	1.202005
F	−0.481135	1.88825	−1.984631
F	−0.378524	4.529097	−1.595739
F	−2.007948	5.74685	0.25034
F	−3.8699	4.247955	1.6182
C	−3.742802	−0.666573	−1.072875
C	−4.696089	0.087993	−1.775371
C	−5.837254	−0.469087	−2.377808
C	−6.054514	−1.84922	−2.30313
C	−5.124549	−2.651495	−1.627924
C	−4.004526	−2.048189	−1.045617

Table S6. *Cont.*

Atomtype	X Coordinates	Y Coordinates	Z Coordinates
F	−4.55567	1.429659	−1.926453
F	−6.720142	0.310629	−3.036208
F	−7.139255	−2.402397	−2.879023
F	−5.317094	−3.985781	−1.554069
F	−3.138821	−2.890805	−0.409957
C	1.120912	−2.759256	−2.50057
C	5.023213	2.334086	−3.087962
C	2.27879	3.28154	3.513158
C	−0.119581	−3.706101	1.232762
C	6.827686	−2.427937	−0.870408
H	4.29639	1.259072	2.528729
H	4.366541	−1.536575	2.557094
H	0.864477	−3.2657	−1.561572
H	0.353448	−2.994397	−3.255382
H	2.087741	−3.171316	−2.83714
H	0.139752	0.579189	−3.969144
H	1.896624	0.834267	−3.943061
H	1.20849	−0.605712	−4.770649
H	−1.418155	−0.474808	−2.527603
H	−1.206676	−1.854813	−1.390873
H	−0.211717	−0.345392	−1.1628
H	3.913645	4.466378	−2.217915
H	2.324846	5.893363	−0.960246
H	1.247515	5.044628	1.107224
H	1.727307	−5.609872	0.469074
H	3.496087	−6.24878	−1.158836
H	5.15362	−4.560568	−1.934995
H	1.64551	1.546486	2.40607
H	−0.380357	2.519582	3.340938
H	−0.161791	4.02888	2.42671
H	−0.517837	2.511703	1.56248
H	1.867005	2.907418	4.4646
H	3.359576	3.063895	3.490657
H	2.158419	4.376899	3.489066
H	4.389239	0.872776	−1.68189
H	5.683685	1.596499	−3.57048
H	4.086442	2.395693	−3.664267
H	5.525641	3.313876	−3.137877
H	6.823214	1.252199	−1.324677
H	6.493218	2.915691	−0.758987
H	5.923252	1.517008	0.194503
H	1.206825	−2.053611	1.585986
H	0.864455	−3.20368	3.7574
H	1.717591	−4.649929	3.139049
H	2.599721	−3.112503	3.360006
H	−0.87531	−3.308111	1.924306
H	−0.417164	−3.415896	0.214944
H	−0.14275	−4.805403	1.301925
H	5.244556	−0.997845	−0.671546
H	6.014386	−0.928013	−3.033471
H	5.381726	−2.575286	−3.307079
H	4.259862	−1.230266	−2.930307
H	7.560865	−1.664538	−1.177048
H	6.941072	−2.608456	0.210139
H	7.06538	−3.363668	−1.401399

Table S7. Cartesian geometry of intermediate (+4.6 kcal/mol) in Figure 3 in Angstrom [Å].

Atomtype	X Coordinates	Y Coordinates	Z Coordinates
N	−0.115138	−0.18523	1.053082
N	0.913488	0.04003	−1.13394
N	−1.272836	0.174323	−1.001533
C	−0.139604	0.025039	−0.246152
C	−0.931432	0.277516	−2.346146
C	0.427001	0.173725	−2.433363
C	−2.610459	0.206661	−0.456725
C	−3.095617	1.431936	0.044977
C	−4.39122	1.429329	0.593096
C	−5.153643	0.256165	0.631584
C	−4.636748	−0.943891	0.123946
C	−3.347427	−0.999262	−0.433419
C	−2.264685	2.712969	0.035284
C	−3.004915	3.871564	−0.670278
C	−1.871731	3.110543	1.477501
C	−2.750203	−2.318114	−0.923804
C	−3.773978	−3.216645	−1.650195
C	−2.106648	−3.075476	0.264833
C	2.288301	−0.10358	−0.735115
C	3.108019	1.051413	−0.73741
C	4.437053	0.88923	−0.306261
C	4.91278	−0.360452	0.119079
C	4.072858	−1.480015	0.109499
C	2.7371	−1.38041	−0.325577
C	2.551038	2.418431	−1.143444
C	1.757847	3.048416	0.029314
C	3.62726	3.40304	−1.643178
C	1.859832	−2.628868	−0.411701
C	2.290765	−3.486443	−1.626617
C	1.892525	−3.470161	0.882169
H	−1.692951	0.412968	−3.10434
H	1.097233	0.182746	−3.284123
H	−4.802366	2.358413	0.990484
H	−6.158038	0.275224	1.058468
H	−5.241647	−1.849929	0.161093
H	−1.334406	2.529059	−0.523453
H	−3.273499	3.601495	−1.70339
H	−2.362228	4.765765	−0.696606
H	−3.929623	4.13552	−0.134066
H	−2.768767	3.332032	2.076919
H	−1.234328	4.00915	1.465158
H	−1.32411	2.291261	1.970092
H	−1.948664	−2.091595	−1.644442
H	−4.544138	−3.590591	−0.957704
H	−3.258545	−4.092138	−2.07454
H	−4.276699	−2.675398	−2.466486
H	−1.353084	−2.45086	0.767729
H	−1.627254	−4.002343	−0.091147
H	−2.878931	−3.347869	1.00241
H	5.108711	1.746948	−0.301525
H	5.947396	−0.460403	0.451894
H	4.459974	−2.450217	0.422906
H	1.840171	2.265047	−1.970068
H	1.296614	3.994819	−0.294206

Table S7. *Cont.*

Atomtype	X Coordinates	Y Coordinates	Z Coordinates
H	2.436002	3.267542	0.869573
H	0.960446	2.38219	0.390054
H	4.295981	3.715617	−0.825369
H	3.139164	4.307821	−2.035181
H	4.239633	2.9605	−2.443617
H	0.816377	−2.327287	−0.575071
H	2.22194	−2.909178	−2.561825
H	1.641351	−4.371918	−1.712741
H	3.331121	−3.828809	−1.510374
H	2.898339	−3.870008	1.08227
H	1.201304	−4.32132	0.788458
H	1.579321	−2.879147	1.760414
Si	0.785859	−0.195086	2.401434
C	2.34113	0.793694	2.743029
H	2.755577	0.519806	3.726396
H	3.101169	0.62524	1.964484
H	2.110318	1.871452	2.749409
C	0.100569	−1.191258	3.830026
H	−0.796374	−1.746037	3.519762
H	−0.162783	−0.526689	4.671705
H	0.85506	−1.902103	4.207558

Table S8. Cartesian geometry of $[MeB(C_6F_5)_3]^-$ in Angstrom [Å].

Atomtype	X Coordinates	Y Coordinates	Z Coordinates
C	−0.085722	2.169722	0.648489
C	−0.55931	1.37916	−0.413168
C	−1.792109	1.813886	−0.935125
C	−2.472881	2.963955	−0.507644
C	−1.93008	3.736503	0.524443
C	−0.726926	3.331193	1.110011
B	0.130953	−0.040907	−0.996766
C	0.260887	−0.0072	−2.649816
F	−2.42865	1.094081	−1.899717
F	−3.653954	3.329334	−1.066283
F	−2.56533	4.852141	0.956464
F	−0.204491	4.059693	2.127782
F	1.045234	1.841627	1.323388
C	−0.90643	−1.204383	−0.39225
C	−1.035707	−1.36691	0.998178
C	−1.935764	−2.245505	1.612308
C	−2.780836	−3.022674	0.809889
C	−2.696823	−2.903752	−0.580206
C	−1.771863	−2.008693	−1.147717
F	−0.238337	−0.650691	1.834528
F	−1.762569	−1.97797	−2.50592
F	−3.510703	−3.656574	−1.362895
F	−3.664489	−3.87981	1.375008
F	−2.003613	−2.359794	2.961922
C	1.70902	−0.267619	−0.497571
C	2.254165	−1.473811	−0.030599
C	3.618443	−1.662904	0.251659
C	4.517064	−0.611783	0.050676
C	4.033036	0.608637	−0.435558
C	2.665135	0.743663	−0.703498
F	1.473902	−2.572649	0.163467

Table S8. *Cont.*

Atomtype	X Coordinates	Y Coordinates	Z Coordinates
F	4.07849	−2.8554	0.707034
F	5.83652	−0.77264	0.313158
F	4.896286	1.634331	−0.643621
F	2.281351	1.956317	−1.189055
H	0.679588	−0.958106	−3.024926
H	0.957138	0.795331	−2.947827
H	−0.688098	0.160422	−3.175773

Table S9. Cartesian geometry of transition state (+21.1 kcal/mol) in Figure 3 in Angstrom [Å].

Atomtype	X Coordinates	Y Coordinates	Z Coordinates
C	2.734114	−3.530242	0.239799
C	3.036251	−2.523765	−0.702911
C	3.113383	−2.746967	−2.098919
C	2.879733	−4.061703	−2.542732
C	2.595863	−5.089419	−1.633623
C	2.511537	−4.824989	−0.261346
N	3.426732	−1.217123	−0.218716
C	2.624506	−0.137891	0.019882
N	3.449998	0.884845	0.407251
C	4.76608	0.420911	0.451111
C	4.749723	−0.881171	0.062085
N	1.27843	−0.066048	−0.064994
Si	−0.033708	−0.788033	−1.08582
C	0.038243	0.035833	−2.758806
C	3.082266	2.276468	0.580343
C	3.210295	2.855819	1.863095
C	2.891767	4.222349	1.984998
C	2.463935	4.964054	0.881513
C	2.370964	4.364691	−0.383259
C	2.703535	3.013012	−0.575
C	3.724399	2.092697	3.083892
C	2.874583	2.33837	4.352248
C	2.744664	2.402958	−1.981388
C	4.211809	2.232196	−2.455788
C	3.46096	−1.630537	−3.083005
C	2.726293	−1.789857	−4.435773
C	2.686955	−3.251566	1.740817
C	4.025156	−3.663889	2.40072
C	−0.16982	−2.642817	−1.143679
C	5.194443	2.492398	3.368166
C	1.971878	3.209451	−3.044769
C	4.987646	−1.560373	−3.334133
C	1.501667	−3.950945	2.442189
Si	0.154592	0.523968	1.206844
C	−0.082697	2.321237	1.567166
C	0.530309	−0.433626	2.771257
N	−1.155656	−0.068354	0.099098
C	−2.494649	0.134691	0.273145
N	−3.199671	1.28735	0.00889

Table S9. *Cont.*

Atomtype	X Coordinates	Y Coordinates	Z Coordinates
C	−4.504086	1.131771	0.466599
C	−4.614687	−0.121073	0.988206
N	−3.377416	−0.735341	0.859748
C	−2.777988	2.437707	−0.779851
C	−2.657662	2.272041	−2.188811
C	−2.21565	3.391138	−2.917843
C	−1.946131	4.614957	−2.290177
C	−2.179938	4.767644	−0.92203
C	−2.634733	3.691836	−0.135162
C	−3.167621	−2.165167	1.034444
C	−2.755117	−2.662128	2.291148
C	−2.604676	−4.058199	2.399064
C	−2.870751	−4.904113	1.314767
C	−3.328172	−4.378416	0.101626
C	−3.506439	−2.993402	−0.066222
C	−3.104768	0.997491	−2.92365
C	−2.705665	0.953947	−4.417675
C	−3.096918	3.968189	1.298605
C	−4.521724	4.586263	1.252454
C	−2.570556	−1.751123	3.505067
C	−1.648733	−2.351244	4.587923
C	−4.168404	−2.443572	−1.333138
C	−5.708165	−2.554223	−1.180713
C	−4.648077	0.83237	−2.875552
C	−2.181147	4.931579	2.091937
C	−3.942272	−1.427346	4.15362
C	−3.749825	−3.137985	−2.648674
H	−5.233815	1.919354	0.345399
H	−5.4591	−0.64875	1.410746
H	5.539259	−1.612019	−0.054335
H	5.577684	1.078223	0.731683
H	0.599745	2.661076	2.359638
H	0.029077	2.967726	0.686802
H	−1.110108	2.436101	1.944327
H	0.346538	−1.508075	2.650688
H	1.601792	−0.293978	3.00247
H	−0.03287	−0.071659	3.642432
H	−0.659449	−0.419339	−3.46783
H	−0.188626	1.109692	−2.698462
H	1.045233	−0.074727	−3.183881
H	0.20131	−3.084095	−0.209097
H	−1.215963	−2.950598	−1.25395
H	0.420375	−3.06646	−1.969089
H	−2.103748	3.317387	−3.997911
H	−1.6005	5.463047	−2.88355
H	−2.039124	5.742491	−0.456198
H	−2.291708	−4.492327	3.347147
H	−2.746688	−5.982438	1.426681

Table S9. *Cont.*

Atomtype	X Coordinates	Y Coordinates	Z Coordinates
H	−3.567485	−5.051816	−0.721163
H	2.98978	4.708978	2.955467
H	2.221339	6.021574	0.998995
H	2.070547	4.970068	−1.237054
H	2.932327	−4.287081	−3.607402
H	2.435099	−6.10461	−2.000107
H	2.285838	−5.636705	0.429946
H	−2.65314	0.125732	−2.420231
H	−2.927988	−0.045545	−4.81906
H	−3.30026	1.680654	−4.991708
H	−1.644655	1.175633	−4.595356
H	−4.928334	−0.116438	−3.358676
H	−5.055981	0.824582	−1.858898
H	−5.12567	1.653157	−3.43183
H	−3.15989	3.015739	1.849668
H	−2.517908	4.968309	3.138918
H	−1.125863	4.627299	2.072112
H	−2.253915	5.95437	1.692001
H	−4.910208	4.713078	2.274677
H	−4.481552	5.575995	0.77225
H	−5.23467	3.975574	0.679354
H	−3.919754	−1.376464	−1.431648
H	−4.285766	−2.663092	−3.484635
H	−2.670874	−3.073686	−2.855659
H	−4.031743	−4.201546	−2.646708
H	−6.206874	−2.1028	−2.052299
H	−6.00522	−3.612379	−1.119148
H	−6.06685	−2.046491	−0.27369
H	−2.133962	−0.802932	3.151857
H	−3.793686	−0.805694	5.050398
H	−4.608508	−0.883906	3.47024
H	−4.443692	−2.358399	4.459601
H	−1.391518	−1.576957	5.326022
H	−2.165421	−3.158952	5.128879
H	−0.719892	−2.764715	4.170425
H	3.154191	−0.672221	−2.631965
H	5.216147	−0.737241	−4.028995
H	5.549799	−1.39078	−2.405021
H	5.339181	−2.501827	−3.78374
H	2.828899	−0.864443	−5.023275
H	3.17523	−2.601253	−5.028343
H	1.653408	−2.011078	−4.305491
H	2.56116	−2.169007	1.890295
H	1.460552	−3.631385	3.495178
H	0.538153	−3.711903	1.965049
H	1.619851	−5.044964	2.435431
H	3.9982	−3.449588	3.480721
H	4.202586	−4.742072	2.265702
H	4.873501	−3.117953	1.958834

Table S9. *Cont.*

Atomtype	X Coordinates	Y Coordinates	Z Coordinates
H	2.285173	1.406269	−1.936788
H	4.224866	1.776879	−3.459167
H	4.702934	3.215279	−2.519953
H	4.80276	1.593833	−1.785712
H	1.88977	2.608826	−3.963065
H	0.959026	3.475951	−2.711818
H	2.506539	4.136899	−3.30274
H	3.697045	1.011565	2.871424
H	5.575152	1.938764	4.240289
H	5.855898	2.291096	2.511717
H	5.254287	3.569218	3.589234
H	3.225175	1.679731	5.161351
H	2.97197	3.376102	4.704407
H	1.810994	2.135836	4.174378

Table S10. Cartesian geometry of **2** in Angstrom [Å].

Atomtype	X Coordinates	Y Coordinates	Z Coordinates
N	1.226643	−0.092927	−0.101329
N	3.358153	−1.257043	−0.445762
N	3.474008	0.869552	0.045041
C	2.587928	−0.159361	−0.158561
C	4.777536	0.41539	−0.144621
C	4.705469	−0.906732	−0.438365
C	3.230811	2.216818	0.514491
C	2.86205	2.397535	1.869944
C	2.698736	3.723989	2.306469
C	2.936881	4.806466	1.446303
C	3.35113	4.586899	0.12913
C	3.506942	3.280774	−0.373095
C	2.751635	1.215381	2.836543
C	4.162134	0.753699	3.285395
C	1.892325	1.507194	4.082188
C	3.962078	3.071249	−1.818806
C	5.439148	3.506435	−1.980027
C	3.07319	3.828459	−2.83357
C	2.975271	−2.637266	−0.64726
C	3.132603	−3.516274	0.448062
C	2.915415	−4.884201	0.199934
C	2.547289	−5.334258	−1.074424
C	2.383951	−4.427528	−2.130746
C	2.607296	−3.052172	−1.94477
C	3.490545	−3.024031	1.850368
C	2.603988	−3.677095	2.936942
C	4.984429	−3.277574	2.165293
C	2.511593	−2.056624	−3.098277
C	3.887269	−1.910143	−3.795081
C	1.427964	−2.427869	−4.131911

Table S10. *Cont.*

Atomtype	X Coordinates	Y Coordinates	Z Coordinates
N	−1.223137	0.047666	−0.064947
N	−3.284937	1.351643	0.197317
N	−3.442627	−0.812076	0.499749
C	−2.554524	0.191415	0.18309
C	−4.711437	−0.266631	0.711066
C	−4.613853	1.069406	0.5228
C	−3.320144	−2.238001	0.273084
C	−3.359776	−2.68975	−1.066391
C	−3.313641	−4.079005	−1.276355
C	−3.279647	−4.972017	−0.199195
C	−3.29995	−4.490476	1.116117
C	−3.32501	−3.109857	1.387957
C	−3.491306	−1.754661	−2.26597
C	−4.818307	−2.008383	−3.018389
C	−2.280313	−1.895727	−3.211531
C	−3.396422	−2.616653	2.831663
C	−4.849225	−2.708913	3.364198
C	−2.457772	−3.407719	3.775524
C	−2.897639	2.725103	−0.044569
C	−3.056503	3.236447	−1.35515
C	−2.848082	4.617441	−1.525731
C	−2.506466	5.435488	−0.440065
C	−2.345727	4.889392	0.838935
C	−2.540745	3.51642	1.069648
C	−3.425943	2.339282	−2.53853
C	−2.829058	2.836697	−3.875253
C	−4.961301	2.198338	−2.69101
C	−2.390692	2.920321	2.468467
C	−3.71668	3.045751	3.259929
C	−1.24421	3.567685	3.276685
H	5.615412	1.091902	−0.03922
H	5.466332	−1.648387	−0.642477
H	2.409892	3.917576	3.338793
H	2.81753	5.826349	1.815452
H	3.554356	5.435082	−0.525365
H	2.278011	0.378606	2.303643
H	4.791821	0.456823	2.434835
H	4.072147	−0.109181	3.964877
H	4.671995	1.564993	3.827346
H	2.38722	2.233017	4.745542
H	1.753973	0.578519	4.655508
H	0.903319	1.906307	3.814772
H	3.897261	1.999706	−2.064106
H	5.548052	4.581622	−1.770293
H	5.778108	3.320166	−3.01062
H	6.102721	2.961261	−1.290522
H	2.021895	3.504666	−2.778809
H	3.439915	3.641321	−3.854428
H	3.104696	4.914562	−2.660188

Table S10. *Cont.*

Atomtype	X Coordinates	Y Coordinates	Z Coordinates
H	3.028527	−5.599193	1.015119
H	2.385976	−6.39962	−1.246401
H	2.094757	−4.794968	−3.114938
H	3.321304	−1.935081	1.892583
H	2.778337	−3.181881	3.904841
H	2.850619	−4.742028	3.062828
H	1.532976	−3.605836	2.689991
H	5.204565	−4.355614	2.12461
H	5.224841	−2.913535	3.176289
H	5.642783	−2.765113	1.448199
H	2.244484	−1.07514	−2.675914
H	4.664039	−1.574992	−3.091011
H	3.820263	−1.175316	−4.61285
H	4.202324	−2.87477	−4.222061
H	1.713735	−3.32112	−4.707978
H	1.303043	−1.602097	−4.848818
H	0.457383	−2.626637	−3.652215
H	−5.551344	−0.907444	0.942837
H	−5.341139	1.867973	0.58401
H	−3.331977	−4.460029	−2.298078
H	−3.256666	−6.047248	−0.383418
H	−3.299543	−5.195184	1.94732
H	−3.517101	−0.713363	−1.916405
H	−5.683947	−1.870638	−2.352587
H	−4.909833	−1.306211	−3.861975
H	−4.853454	−3.032464	−3.420324
H	−2.235302	−2.905986	−3.647079
H	−2.352897	−1.171945	−4.038307
H	−1.344653	−1.713192	−2.665104
H	−3.099703	−1.555161	2.851419
H	−5.19593	−3.753619	3.339099
H	−4.891306	−2.355466	4.40615
H	−5.548199	−2.104044	2.767929
H	−1.441239	−3.507059	3.364111
H	−2.398124	−2.900262	4.750234
H	−2.846993	−4.421032	3.955933
H	−2.955747	5.056928	−2.516682
H	−2.359811	6.505623	−0.594732
H	−2.067944	5.538025	1.669072
H	−3.014693	1.335233	−2.338463
H	−2.936121	2.053677	−4.641118
H	−3.366418	3.725914	−4.238242
H	−1.764475	3.101792	−3.782746
H	−5.415314	3.187239	−2.85903
H	−5.19265	1.561863	−3.559613
H	−5.42732	1.7475	−1.804142
H	−2.159019	1.847106	2.361006
H	−4.544086	2.524035	2.757435
H	−3.597816	2.614751	4.266488

Table S10. *Cont.*

Atomtype	X Coordinates	Y Coordinates	Z Coordinates
H	−3.992789	4.106097	3.36918
H	−1.482845	4.608397	3.543906
H	−1.103774	3.01436	4.217541
H	−0.292875	3.567374	2.722426
Si	−0.042148	−1.14751	0.646875
Si	0.053598	1.054528	−0.884299
C	0.212714	2.846564	−0.414124
H	−0.568563	3.444875	−0.897973
H	0.178471	3.021907	0.667137
H	1.173811	3.219401	−0.784411
C	0.099658	0.831432	−2.748569
H	0.183043	−0.222853	−3.042454
H	−0.796577	1.250575	−3.229001
H	0.970497	1.371683	−3.159748
C	−0.040518	−0.992425	2.514037
H	0.830128	−1.498438	2.959724
H	−0.00538	0.066651	2.806774
H	−0.939988	−1.444626	2.953592
C	−0.140923	−2.882114	−0.032169
H	−0.082826	−2.883815	−1.128853
H	0.686615	−3.494499	0.346149
H	−1.070822	−3.376387	0.266982

Table S11. Cartesian geometry of **3** in Angstrom [Å].

Atomtype	X Coordinates	Y Coordinates	Z Coordinates
C	−0.001022	−0.584754	0.026992
N	1.111584	−1.452273	−0.044649
C	0.674742	−2.773582	−0.150749
C	−0.682626	−2.771764	−0.150863
N	−1.115914	−1.449287	−0.04482
C	2.528832	−1.000638	0.042638
C	3.469795	−2.217125	−0.042164
C	−2.531935	−0.993834	0.042654
C	−3.476045	−2.20784	−0.042525
N	0.000362	0.684881	0.160725
Si	0.003788	2.360565	−0.014021
C	0.002962	2.841442	−1.866343
C	−2.753519	−0.286564	1.39721
C	−2.837782	−0.050849	−1.141034
C	1.542737	3.160611	0.791672
C	2.837124	−0.058947	−1.141465
C	2.752245	−0.29356	1.396995
H	1.359229	−3.60656	−0.228413
H	−1.369407	−3.602867	−0.228644
H	2.170603	0.809177	−1.128744
H	3.880563	0.287606	−1.071768
H	2.706274	−0.595965	−2.094234
H	3.790176	0.071373	1.453573

Table S11. *Cont.*

Atomtype	X Coordinates	Y Coordinates	Z Coordinates
H	2.06685	0.553853	1.505409
H	2.581993	−1.003108	2.222271
H	4.505728	−1.853964	0.023184
H	3.297645	−2.918923	0.788738
H	3.35161	−2.752881	−0.997041
H	−2.06653	0.559589	1.50553
H	−3.790752	0.080304	1.454169
H	−2.584364	−0.99656	2.222327
H	−3.88037	0.298238	−1.071235
H	−2.169065	0.815602	−1.127721
H	−2.708177	−0.587701	−2.094065
H	−4.511052	−1.842053	0.022853
H	−3.35918	−2.743642	−0.997537
H	−3.305789	−2.910334	0.788191
H	0.00554	3.938778	−1.991405
H	0.890429	2.443913	−2.38771
H	−0.887978	2.448465	−2.385241
H	1.514305	4.259547	0.685654
H	1.601496	2.933236	1.869974
H	2.47681	2.80232	0.327212
C	−1.531405	3.165573	0.793857
H	−1.499402	4.264511	0.689155
H	−2.467095	2.810886	0.32985
H	−1.589963	2.936994	1.871929

Table S12. Cartesian geometry of transition state (+11.3 kcal/mol) in Figure 4 in Angstrom [Å].

Atomtype	X Coordinates	Y Coordinates	Z Coordinates
N	−3.812657	−1.162689	0.228657
C	−4.653869	−0.490288	1.109699
C	−4.868576	0.756462	0.618833
N	−4.178404	0.869885	−0.583893
C	−3.514009	−0.326076	−0.847795
C	−4.163396	2.087184	−1.459982
C	−2.73439	2.665474	−1.51147
N	−2.817506	−0.640346	−1.901912
Si	−1.463165	−0.638479	−2.826215
C	−0.906918	−2.345753	−3.435449
C	−3.481682	−2.628557	0.31566
C	−4.226677	−3.353154	−0.825395
C	−4.683373	1.703573	−2.862118
C	−5.09802	3.162735	−0.869919
C	−1.233807	0.705196	−4.145157
C	−1.96283	−2.862613	0.207739
C	−3.952001	−3.17548	1.679076
H	−4.982236	−0.92247	2.04283
H	−5.431649	1.573402	1.045875
H	−3.90651	−2.956548	−1.798582
H	−4.002103	−4.43031	−0.78268

Table S12. *Cont.*

Atomtype	X Coordinates	Y Coordinates	Z Coordinates
H	−5.314168	−3.214046	−0.719926
H	−1.581441	−2.553529	−0.767017
H	−1.421703	−2.330948	0.99762
H	−1.770204	−3.94067	0.320067
H	−3.656773	−4.232537	1.738434
H	−3.476077	−2.638099	2.513689
H	−5.046211	−3.122301	1.786815
H	−2.023517	1.961906	−1.945825
H	−2.736825	3.573825	−2.133543
H	−2.386717	2.924976	−0.506592
H	−4.626645	2.58484	−3.519751
H	−4.087437	0.891879	−3.293538
H	−5.733545	1.377658	−2.799729
H	−5.091629	4.024383	−1.552399
H	−6.133048	2.797564	−0.785312
H	−4.749653	3.506218	0.116126
H	−0.757313	1.604495	−3.726607
H	−0.59248	0.333907	−4.961884
H	−2.203397	1.001393	−4.576703
H	−1.788024	−2.998835	−3.550041
H	−0.387397	−2.290943	−4.405997
H	−0.234723	−2.812285	−2.698658
C	0.17831	−0.07596	−1.416633
H	0.790464	0.136565	−2.298165
H	−0.584433	0.658807	−1.167464
H	−0.032365	−1.121954	−1.197936
B	1.315759	0.132708	−0.072807
C	2.58264	−0.804676	−0.556392
C	1.659531	1.72393	0.174966
C	0.525644	−0.390574	1.291799
C	2.443188	2.056091	1.295318
C	2.739587	3.366761	1.685959
C	2.235307	4.433705	0.928391
C	1.457776	4.161046	−0.202127
C	1.199191	2.827007	−0.556103
F	2.971049	1.062764	2.053604
F	3.499462	3.616212	2.770614
F	2.498705	5.704972	1.283261
F	0.968491	5.178597	−0.942456
F	0.443512	2.669799	−1.688297
C	1.013975	−1.354936	2.192476
C	0.293947	−1.818141	3.306287
C	−0.957868	−1.264739	3.594951
C	−1.456744	−0.244988	2.777034
C	−0.719411	0.155359	1.655009
F	2.238174	−1.903237	2.030337
F	0.799116	−2.776365	4.10699
F	−1.684476	−1.707827	4.640494

Table S12. *Cont.*

Atomtype	X Coordinates	Y Coordinates	Z Coordinates
F	−2.648923	0.318334	3.072585
F	−1.306347	1.130346	0.894047
C	3.860335	−0.330756	−0.900612
C	4.88593	−1.152199	−1.401999
C	4.651355	−2.518195	−1.591393
C	3.38972	−3.041904	−1.278289
C	2.407773	−2.183154	−0.775923
F	4.177041	0.981766	−0.786509
F	6.091069	−0.634187	−1.711484
F	5.618318	−3.318362	−2.075983
F	3.139681	−4.354496	−1.463112
F	1.20062	−2.772028	−0.4975

Table S13. Cartesian geometry of intermediate (+4.5 kcal/mol) in Figure 4 in Angstrom [Å].

Atomtype	X Coordinates	Y Coordinates	Z Coordinates
N	−1.107007	−1.274014	−0.002194
C	−0.678692	−2.587186	−0.006976
C	0.688627	−2.584751	−0.006876
N	1.112422	−1.269979	−0.003268
C	0.001209	−0.440502	0.001188
C	2.557843	−0.816943	−0.000676
C	2.817396	0.00351	−1.280705
N	−0.003682	0.872216	0.004833
Si	−0.00417	2.473453	−0.003373
C	−1.594766	3.471766	−0.014669
C	−2.553224	−0.825075	0.003207
C	−2.811637	−0.014449	1.289993
C	2.814743	−0.003258	1.284419
C	3.481165	−2.049821	−0.002562
C	1.577291	3.486916	0.01108
C	−2.816668	−0.002251	−1.274691
C	−3.47281	−2.060733	−0.000422
H	−1.364008	−3.422971	−0.008931
H	1.376857	−3.417981	−0.013145
H	−2.154758	0.86049	1.356466
H	−3.857517	0.326707	1.289086
H	−2.652665	−0.64601	2.177191
H	−3.864252	0.333895	−1.269051
H	−2.163694	0.876347	−1.332449
H	−2.655811	−0.623697	−2.1687
H	−4.511746	−1.703974	0.00225
H	−3.325541	−2.673903	−0.90218
H	−3.323716	−2.680426	0.896561
H	2.156292	0.8754	−1.344495
H	3.861761	0.349802	−1.273402
H	2.665105	−0.622513	−2.172941
H	3.859537	0.341546	1.281782

Table S13. *Cont.*

Atomtype	X Coordinates	Y Coordinates	Z Coordinates
H	2.155407	0.869738	1.351111
H	2.659016	−0.634096	2.172678
H	4.518874	−1.68935	0.000047
H	3.33359	−2.669261	0.89479
H	3.337011	−2.664174	−0.904035
H	1.582983	4.199054	−0.845665
H	1.62998	4.101946	0.939945
H	2.47482	2.843831	−0.04347
H	−1.63784	4.123088	−0.905096
H	−2.481011	2.822042	−0.008161
H	−1.635288	4.135215	0.86758

Table S14. Cartesian geometry of transition state (+12.6 kcal/mol) in Figure 4 in Angstrom [Å].

Atomtype	X Coordinates	Y Coordinates	Z Coordinates
N	3.553355	−0.293631	−0.7134
C	2.457163	0.32102	−0.130363
N	2.774961	1.671575	−0.061764
C	4.017428	1.873481	−0.639792
C	4.489329	0.669651	−1.049395
N	1.283887	−0.281914	0.25103
Si	1.116582	−0.866792	1.960753
C	0.757087	−2.740291	2.068334
C	2.085831	2.800956	0.698696
C	0.570214	2.787803	0.468338
C	3.818798	−1.75884	−1.038669
C	3.05911	−2.68676	−0.079777
Si	−0.441014	−0.478514	−1.355813
C	0.18831	0.644559	−2.759413
N	−1.938655	−0.076991	−0.747012
C	−3.215909	−0.040053	−0.514267
N	−4.036672	1.096825	−0.550994
C	−5.323954	0.773064	−0.129571
C	−5.357274	−0.555329	0.130051
N	−4.089811	−1.077956	−0.110668
C	−3.638252	2.47996	−0.964274
C	−3.040218	3.17953	0.278038
C	−3.824188	−2.550266	−0.212359
C	−5.030105	−3.352456	0.317005
C	−0.416425	−2.256079	−2.054715
C	−2.607377	−2.884831	0.666248
C	−3.626039	−2.901458	−1.704791
C	−2.64179	2.414016	−2.151569
C	−4.88813	3.262545	−1.434853
C	−0.31269	0.049069	2.806981
C	2.795549	−0.591893	2.838009
C	2.442284	2.679484	2.214644
C	2.608433	4.157091	0.160804
C	3.436894	−1.992753	−2.519196

Table S14. *Cont.*

Atomtype	X Coordinates	Y Coordinates	Z Coordinates
C	5.32861	−2.0458	−0.835806
H	−6.104825	1.512118	−0.023567
H	−6.177366	−1.161803	0.486765
H	5.4148	0.436863	−1.554395
H	4.481541	2.844732	−0.716677
H	−5.934623	−3.182741	−0.285746
H	−4.773528	−4.419801	0.25019
H	−5.242254	−3.116463	1.371194
H	−2.288121	−3.923817	0.494955
H	−1.778724	−2.213956	0.437647
H	−2.863947	−2.769057	1.730645
H	−2.911737	−2.219601	−2.173692
H	−3.254795	−3.931887	−1.806942
H	−4.588283	−2.822157	−2.234183
H	−2.307739	2.524289	0.765097
H	−2.54695	4.119155	−0.011275
H	−3.834323	3.413068	1.004511
H	−1.617662	2.24616	−1.81288
H	−2.901634	1.591394	−2.830883
H	−2.681929	3.358013	−2.71603
H	−5.60682	3.436381	−0.621071
H	−4.553772	4.247782	−1.790169
H	−5.394559	2.743469	−2.26263
H	3.26041	−2.408092	0.9644
H	1.985188	−2.675919	−0.254931
H	3.431822	−3.710094	−0.233786
H	3.329135	−3.07166	−2.705695
H	2.488036	−1.502797	−2.76966
H	4.218298	−1.604945	−3.19087
H	5.48457	−3.119819	−1.010388
H	5.972854	−1.513154	−1.5482
H	5.644601	−1.809786	0.191436
H	0.114349	1.813885	0.655996
H	0.125603	3.516371	1.159341
H	0.334298	3.112869	−0.552329
H	1.573087	2.368123	2.808673
H	3.247193	1.955407	2.383906
H	2.785687	3.650517	2.602032
H	1.984737	4.943844	0.607746
H	3.647271	4.359076	0.460344
H	2.516973	4.219926	−0.933874
H	1.640108	−3.265081	2.466753
H	−0.083598	−2.930866	2.753463
H	0.500884	−3.212719	1.111103
H	−1.281096	−0.300028	2.413031
H	−0.294414	−0.16328	3.890423
H	−0.276317	1.140558	2.677177
H	3.200467	−1.565508	3.158595

Table S14. *Cont.*

Atomtype	X Coordinates	Y Coordinates	Z Coordinates
H	3.564599	−0.115267	2.210392
H	2.68035	0.026412	3.74293
H	−0.832306	−2.987925	−1.349651
H	−1.068497	−2.27049	−2.944252
H	0.564423	−2.621983	−2.386376
H	0.358169	1.6903	−2.465951
H	1.135026	0.265821	−3.180973
H	−0.554261	0.647325	−3.574491

Table S15. Cartesian geometry of **4** in Angstrom [Å].

Atomtype	X Coordinates	Y Coordinates	Z Coordinates
N	−3.705636	1.084185	−0.598193
C	−2.849083	0.009582	−0.282324
N	−3.716042	−1.093657	−0.155667
C	−5.015687	−0.703982	−0.487444
C	−5.011276	0.62374	−0.742378
N	−1.547756	0.086813	−0.125618
Si	−0.12952	−0.296337	−0.952636
C	−0.262698	−1.957152	−1.890605
C	−3.47762	−2.48537	0.365915
C	−2.186863	−2.571487	1.187526
C	−3.350036	2.542332	−0.523853
C	−2.294757	2.869334	−1.592918
N	1.286862	−0.334011	0.245559
Si	1.312729	−1.127485	1.887353
C	0.040653	−0.29326	3.016189
C	2.433379	0.331045	−0.117088
N	2.727126	1.665636	0.111393
C	3.973334	1.9476	−0.427063
C	4.456304	0.811258	−0.986707
N	3.523192	−0.194223	−0.788213
C	2.009245	2.732092	0.941645
C	2.555101	2.653732	2.381756
C	3.802627	−1.578886	−1.369697
C	3.543907	−1.498046	−2.887666
C	2.335585	4.122504	0.34116
C	0.486364	2.575308	0.929169
C	5.288847	−1.924568	−1.10047
C	2.952795	−2.681657	−0.729555
C	3.082143	−0.935684	2.564773
C	0.947325	−2.998613	1.803296
C	0.495627	0.914319	−2.29414
C	−3.443603	−3.461079	−0.828181
C	−4.642603	−2.871421	1.316288
C	−4.595277	3.410617	−0.794515
C	−2.848156	2.834841	0.906413
H	−5.827642	1.2702	−1.030407

Table S15. *Cont.*

Atomtype	X Coordinates	Y Coordinates	Z Coordinates
H	−5.83355	−1.409048	−0.528188
H	5.383982	0.647001	−1.51488
H	4.425933	2.926345	−0.374116
H	−5.592258	−3.021046	0.783829
H	−4.389111	−3.823558	1.805077
H	−4.784399	−2.10324	2.090977
H	−2.106336	−3.594683	1.582812
H	−1.306518	−2.363297	0.583175
H	−2.206961	−1.868047	2.030545
H	−2.60673	−3.232081	−1.499728
H	−3.330146	−4.492591	−0.459493
H	−4.382185	−3.399643	−1.400105
H	−2.033212	2.154016	1.17342
H	−2.490538	3.874302	0.967647
H	−3.673277	2.705674	1.624088
H	−1.416179	2.240872	−1.45368
H	−2.702912	2.692798	−2.600376
H	−2.004809	3.928605	−1.51249
H	−5.381734	3.244053	−0.043426
H	−4.286419	4.464148	−0.736398
H	−5.005577	3.231058	−1.80019
H	3.212731	−2.818822	0.326857
H	1.885939	−2.486593	−0.812582
H	3.184712	−3.61765	−1.258637
H	3.832262	−2.453843	−3.35003
H	2.482578	−1.314824	−3.09979
H	4.14173	−0.696475	−3.347065
H	5.442377	−2.980622	−1.363478
H	5.985015	−1.334095	−1.712484
H	5.536903	−1.789955	−0.037246
H	0.142669	1.597276	1.262201
H	0.080106	3.337233	1.609676
H	0.075307	2.768358	−0.066996
H	2.220538	1.740899	2.888738
H	3.655356	2.68872	2.388162
H	2.177564	3.518632	2.947994
H	1.664369	4.854236	0.811566
H	3.364911	4.448666	0.550491
H	2.159231	4.138102	−0.744777
H	1.848	−3.565603	2.090038
H	0.146735	−3.264559	2.509999
H	0.641101	−3.353395	0.810228
H	−0.901106	−0.141364	2.466382
H	−0.163884	−0.93592	3.889282
H	0.370974	0.686809	3.393245
H	3.787391	−1.611586	2.055297
H	3.493302	0.081493	2.496507
H	3.078849	−1.217085	3.63206

Table S15. *Cont.*

Atomtype	X Coordinates	Y Coordinates	Z Coordinates
H	−0.35226	−2.849023	−1.252504
H	−1.178006	−1.906533	−2.505561
H	0.578263	−2.126057	−2.581528
H	0.70659	1.933336	−1.939766
H	1.419404	0.534594	−2.760787
H	−0.261665	0.991564	−3.091606

Table S16. Cartesian geometry of **7** in Angstrom [Å].

C	2.580495	−0.644357	−0.310905
N	1.866245	−1.793626	−0.509466
C	2.558904	−2.967458	−0.598436
C	3.932135	−3.041877	−0.491114
C	4.700112	−1.850764	−0.276572
C	3.955212	−0.632784	−0.193175
Si	−0.048535	−1.763454	−0.638905
C	−0.358348	−2.402119	−2.385961
N	6.051767	−1.877981	−0.15851
C	6.779121	−3.152823	−0.246579
N	−0.351345	−0.153654	−0.373647
C	−1.133238	0.734611	0.145184
N	−0.73616	1.996757	0.558955
C	−1.83657	2.719227	1.029893
C	−2.927221	1.913236	0.919377
N	−2.500742	0.697048	0.3702
C	0.623208	2.439527	0.433636
C	1.079928	2.844162	−0.840625
C	2.440643	3.183185	−0.963445
C	3.299353	3.126644	0.140901
C	2.815811	2.722822	1.394484
C	1.466797	2.364458	1.567855
C	0.166956	2.891303	−2.062827
C	0.584663	1.818124	−3.095339
C	0.944316	1.83311	2.902437
C	1.649305	2.445413	4.129914
C	−3.307994	−0.453268	0.076388
C	−3.74624	−1.262975	1.148761
C	−4.512744	−2.400548	0.83047
C	−4.802113	−2.72291	−0.499652
C	−4.348931	−1.903136	−1.543945
C	−3.599533	−0.74243	−1.28235
C	−3.447437	−0.911019	2.605495
C	−3.024855	−2.132595	3.452029
C	−3.150596	0.201293	−2.402578
C	−4.015096	1.487782	−2.406059
C	−0.58448	−3.014899	0.668684
C	−4.682086	−0.228725	3.244457
C	−3.183422	−0.429718	−3.809234

Table S16. *Cont.*

C	1.055868	0.287538	2.927173
C	0.150724	4.298025	−2.703208
C	6.795417	−0.628311	0.058161
H	3.494048	2.688487	2.247394
H	2.114936	−0.016583	2.880601
H	0.622083	−0.110657	3.858993
H	0.532757	−0.158097	2.068859
H	6.481154	−0.146673	0.998967
H	7.865893	−0.852665	0.117526
H	6.438938	−3.850197	0.536558
H	−0.277549	−2.693672	1.676633
H	−1.730342	3.735992	1.387943
H	4.34855	3.409906	0.029032
H	2.822559	3.500208	−1.935092
H	1.629487	3.545822	4.098883
H	1.143134	2.111677	5.048676
H	2.700115	2.11984	4.195178
H	1.146582	4.572245	−3.08641
H	−0.553703	4.314383	−3.550009
H	−0.15934	5.063524	−1.974872
H	−3.96741	2.079472	1.167639
H	−4.876274	−3.041861	1.634129
H	−5.388847	−3.614961	−0.727261
H	−4.590851	−2.166982	−2.573102
H	−2.615013	−0.191082	2.624916
H	−5.540448	−0.919468	3.244232
H	−4.464839	0.054688	4.286769
H	−4.976036	0.676266	2.690835
H	−2.168752	−2.657604	3.003986
H	−2.74286	−1.797447	4.462557
H	−3.851966	−2.851345	3.561096
H	−2.108623	0.490611	−2.199117
H	−4.217361	−0.633497	−4.13115
H	−2.744272	0.275643	−4.531234
H	−2.614843	−1.369821	−3.855098
H	−3.962539	2.021658	−1.446989
H	−3.666035	2.169433	−3.198841
H	−5.068311	1.235475	−2.608746
H	−0.124767	2.085466	2.975464
H	−0.860034	2.662108	−1.744015
H	0.556257	0.818823	−2.63692
H	−0.103154	1.835604	−3.95641
H	1.603686	2.011575	−3.468991
H	1.991645	0.267644	−0.245037
H	4.438211	0.327195	−0.033261
H	4.407457	−4.015946	−0.572804
H	1.962358	−3.865197	−0.758799
H	6.626618	0.074365	−0.774431
H	6.627491	−3.622083	−1.23276

Table S16. *Cont.*

H	−1.683418	−3.098564	0.659729
H	−0.170229	−4.021264	0.487743
H	−0.085252	−1.650081	−3.14286
H	0.203151	−3.327373	−2.599288
H	−1.430715	−2.626813	−2.500938
H	7.849428	−2.966105	−0.10772

Table S17. Cartesian geometry of transition state (+26.7 kcal/mol) in Figure S7 in Angstrom [Å].

Atomtype	X Coordinates	Y Coordinates	Z Coordinates
C	−2.637825	−2.264143	−2.786737
C	−2.99308	−2.248879	−1.41646
C	−3.138091	−3.430475	−0.647492
C	−2.855922	−4.652158	−1.287536
C	−2.475558	−4.696928	−2.631539
C	−2.376539	−3.513468	−3.372589
N	−3.351639	−0.986392	−0.795468
C	−2.542748	0.055212	−0.376329
N	−3.416015	0.974858	0.19969
C	−4.726173	0.518778	0.08407
C	−4.685869	−0.694397	−0.519837
N	−1.211135	0.20417	−0.584055
Si	0.528893	−1.376853	0.040691
C	0.735084	−2.697197	−1.301565
C	−3.06832	2.090431	1.065197
C	−3.38809	3.416458	0.685539
C	−2.917837	4.451908	1.517251
C	−2.223826	4.179465	2.698276
C	−2.078567	2.854838	3.129784
C	−2.527453	1.782434	2.341965
C	−4.364934	3.766853	−0.435533
C	−3.952847	5.005249	−1.261487
C	−2.635061	0.376167	2.928984
C	−4.086081	0.156771	3.434916
C	−3.647021	−3.453946	0.795361
C	−2.834005	−4.405979	1.706817
C	−2.566705	−0.976241	−3.600339
C	−3.98395	−0.501208	−4.003575
Si	−0.622583	1.665306	−1.556635
C	−2.093996	2.32062	−2.570922
C	0.589422	1.073377	−2.881777
C	0.052623	2.952065	−0.350825
C	−0.057787	−2.203641	1.626382
N	1.936797	−0.447028	0.099778
C	3.169309	−0.372868	0.468544
N	3.779225	−0.820825	1.647168
C	5.13529	−0.479242	1.669707
C	5.413704	0.147579	0.500952
N	4.228457	0.217282	−0.240014

Table S17. *Cont.*

Atomtype	X Coordinates	Y Coordinates	Z Coordinates
C	3.098114	−1.384475	2.772242
C	2.901123	−2.785562	2.816983
C	2.284047	−3.313157	3.963199
C	1.882024	−2.476701	5.015246
C	2.093313	−1.09503	4.942818
C	2.711101	−0.517841	3.817253
C	4.227218	0.598093	−1.626975
C	4.25651	1.971848	−1.953881
C	4.364469	2.317601	−3.314331
C	4.420221	1.332294	−4.305369
C	4.366815	−0.02285	−3.954788
C	4.275081	−0.421361	−2.610042
C	4.193029	3.058804	−0.882878
C	3.360057	4.280702	−1.330686
C	4.257198	−1.901296	−2.22575
C	5.686736	−2.39953	−1.901051
C	3.37359	−3.673027	1.662682
C	2.63351	−5.022796	1.552658
C	2.985717	0.984776	3.768116
C	3.93173	1.40419	4.916653
C	5.613099	3.524497	−0.478069
C	3.619194	−2.804269	−3.302238
C	4.895756	−3.942541	1.771061
C	1.682634	1.810987	3.797796
C	−5.762311	4.018229	0.193245
C	−1.658774	0.085784	4.081722
C	−5.136323	−3.881441	0.834602
C	−1.674948	−1.078502	−4.852622
H	5.754515	−0.714139	2.526609
H	6.341446	0.532543	0.098575
H	−5.469962	−1.381182	−0.806385
H	−5.552536	1.107622	0.457639
H	1.158386	1.93226	−3.276199
H	1.32324	0.353909	−2.50935
H	0.034495	0.626981	−3.72154
H	1.048465	2.652304	0.004951
H	0.135153	3.943096	−0.825664
H	−0.606451	3.053275	0.523125
H	−2.072942	1.874456	−3.577021
H	−3.081484	2.117904	−2.145181
H	−2.001197	3.409615	−2.69864
H	0.70193	−2.259541	−2.309917
H	1.725222	−3.167549	−1.181811
H	−0.022451	−3.491488	−1.245994
H	0.14599	−1.595411	2.513959
H	−1.126583	−2.446754	1.595453
H	0.48817	−3.146792	1.752999

Table S17. *Cont.*

Atomtype	X Coordinates	Y Coordinates	Z Coordinates
H	4.396725	3.370188	−3.59738
H	4.500987	1.619757	−5.355607
H	4.401278	−0.780501	−4.737456
H	2.110203	−4.387065	4.035149
H	1.405577	−2.908243	5.897891
H	1.784938	−0.45212	5.76909
H	−3.122199	5.485629	1.238536
H	−1.850417	4.999113	3.31483
H	−1.631614	2.652185	4.101149
H	−2.948434	−5.578639	−0.720533
H	−2.262031	−5.656327	−3.106164
H	−2.08617	−3.56222	−4.420908
H	−4.465381	2.91105	−1.114715
H	−6.502928	4.214068	−0.598523
H	−6.108091	3.161775	0.793845
H	−5.725607	4.89404	0.859741
H	−4.599587	5.092227	−2.147841
H	−4.074594	5.927585	−0.672354
H	−2.907382	4.948298	−1.591532
H	−2.125148	−0.211676	−2.952586
H	−3.91708	0.45062	−4.553923
H	−4.463219	−1.246609	−4.658097
H	−4.628441	−0.344438	−3.126242
H	−1.561851	−0.079814	−5.301509
H	−0.674146	−1.464621	−4.60567
H	−2.124527	−1.73378	−5.615474
H	−3.578317	−2.438919	1.215516
H	−5.501192	−3.875919	1.874038
H	−5.780028	−3.217055	0.239936
H	−5.2466	−4.901928	0.435112
H	−3.134024	−4.251654	2.754996
H	−3.037403	−5.458514	1.456158
H	−1.752867	−4.233226	1.622901
H	−2.447848	−0.353437	2.134723
H	−1.712827	−0.980278	4.346402
H	−0.622112	0.318212	3.814049
H	−1.924119	0.667437	4.979453
H	−4.189955	−0.865499	3.834287
H	−4.315955	0.869435	4.242811
H	−4.82742	0.290996	2.634872
H	3.711243	2.625533	0.00741
H	3.158065	4.930005	−0.46471
H	3.904089	4.881295	−2.077178
H	2.401379	3.972024	−1.770836
H	5.549436	4.324939	0.277098
H	6.206745	2.701328	−0.055582
H	6.146273	3.920124	−1.357548
H	3.64957	−2.005224	−1.316081

Table S17. *Cont.*

Atomtype	X Coordinates	Y Coordinates	Z Coordinates
H	3.486928	−3.819735	−2.897595
H	2.635398	−2.424165	−3.616538
H	4.264218	−2.883889	−4.191648
H	5.65787	−3.460228	−1.601733
H	6.331808	−2.308089	−2.7899
H	6.137565	−1.823386	−1.080508
H	3.492002	1.218674	2.821452
H	4.154829	2.480937	4.84841
H	3.469551	1.210882	5.89808
H	4.880619	0.84677	4.87178
H	1.91748	2.886676	3.748888
H	1.036128	1.561068	2.944125
H	1.120086	1.627308	4.726973
H	3.199998	−3.117333	0.72619
H	2.954456	−5.537125	0.633725
H	2.878001	−5.679898	2.402579
H	1.540064	−4.906295	1.515211
H	5.22848	−4.567552	0.926509
H	5.475105	−3.009907	1.755183
H	5.118424	−4.478941	2.70757

Table S18. Cartesian geometry of product (−15.8 kcal/mol) in Figure S7 in Angstrom [Å].

Atomtype	X Coordinates	Y Coordinates	Z Coordinates
N	−3.435579	0.366254	0.935022
C	−2.487463	−0.480443	0.386674
N	−3.205501	−1.390384	−0.375693
C	−4.569261	−1.147069	−0.236019
C	−4.710678	−0.060962	0.565931
N	−1.160157	−0.527123	0.654617
Si	−0.520094	−2.046324	1.488546
C	0.727415	−1.529152	2.80619
C	−2.686074	−2.189505	−1.478605
C	−3.239515	1.614748	1.657659
Si	0.050139	0.844332	0.388233
C	−0.766265	1.977601	−0.871464
N	1.496582	0.039169	−0.024578
C	2.708193	0.493736	−0.132101
N	3.17213	1.67862	−0.729924
C	4.571236	1.741441	−0.666202
C	4.999056	0.627245	−0.023257
N	3.874027	−0.141642	0.29587
C	2.407645	2.412402	−1.699505
C	3.942012	−1.38043	1.020889
C	0.331862	1.815837	1.994109
C	0.341684	−3.148784	0.234596
C	−1.974926	−2.84636	2.437526
H	5.112309	2.573482	−1.09967

Table S18. *Cont.*

Atomtype	X Coordinates	Y Coordinates	Z Coordinates
H	5.991384	0.293556	0.251379
H	−5.589577	0.458622	0.920935
H	−5.297598	−1.76943	−0.739243
H	1.175958	−2.44089	3.237392
H	1.545431	−0.941818	2.375894
H	0.258385	−0.963712	3.625051
H	1.122696	−2.530428	−0.227905
H	0.837791	−3.990626	0.74622
H	−0.305044	−3.544198	−0.558383
H	−1.829809	−2.661757	3.514055
H	−2.968428	−2.452565	2.179177
H	−1.998638	−3.935667	2.291601
H	0.403082	1.157264	2.870578
H	1.288972	2.358019	1.910376
H	−0.457545	2.556804	2.184213
H	−0.447376	1.76832	−1.897754
H	−1.856589	1.911226	−0.819284
H	−0.494937	3.018274	−0.653562
C	3.833529	−2.596559	0.301799
C	3.944453	−3.789844	1.035979
C	4.169634	−3.77011	2.420315
C	4.283994	−2.553464	3.100533
C	4.164626	−1.3299	2.415568
C	3.637184	−2.596599	−1.216896
H	3.859407	−4.747028	0.522
H	4.260787	−4.710538	2.967818
H	4.462516	−2.548016	4.176615
C	4.312172	−0.005259	3.164948
C	1.962076	3.715979	−1.388194
C	1.226137	4.404134	−2.370521
C	0.933618	3.806573	−3.602275
C	1.405577	2.518996	−3.8913
C	2.17159	1.801924	−2.955979
C	2.307646	4.382991	−0.05942
H	0.87371	5.415653	−2.163706
H	0.350495	4.352358	−4.346963
H	1.200053	2.07085	−4.864887
C	2.758638	0.440317	−3.32905
C	−2.680096	−3.59882	−1.390492
C	−2.128465	−4.312362	−2.472586
C	−1.631816	−3.651589	−3.599066
C	−1.761277	−2.261311	−3.705942
C	−2.32533	−1.501105	−2.667506
C	−3.346177	−4.364037	−0.252942
H	−2.096305	−5.400902	−2.42492
H	−1.186606	−4.222478	−4.415919
H	−1.44703	−1.760073	−4.620724
C	−2.713073	−0.043255	−2.908338

Table S18. *Cont.*

Atomtype	X Coordinates	Y Coordinates	Z Coordinates
C	−3.557657	2.826319	0.98788
C	−3.384801	4.022088	1.710051
C	−2.951454	4.015066	3.038784
C	−2.697536	2.80067	3.686475
C	−2.839868	1.574908	3.014558
C	−4.165171	2.89847	−0.41702
H	−3.609575	4.970121	1.221265
H	−2.825943	4.956622	3.576373
H	−2.378968	2.804664	4.727755
C	−2.62338	0.249406	3.73363
H	−3.519359	−3.673575	0.579405
C	−4.729741	−4.874451	−0.730206
C	−2.501454	−5.546753	0.26884
H	−5.244756	−5.397348	0.091307
H	−5.371248	−4.046995	−1.073921
H	−4.611097	−5.577391	−1.569767
H	−2.980118	−5.976226	1.162707
H	−2.429845	−6.347627	−0.483555
H	−1.482429	−5.229928	0.530791
H	−2.119619	−0.416229	3.027509
C	−1.730681	0.348344	4.98537
C	−3.983566	−0.388466	4.106987
H	−3.820945	−1.366317	4.586565
H	−4.525395	0.259637	4.814108
H	−4.618813	−0.541622	3.221765
H	−1.503593	−0.665093	5.35028
H	−0.78336	0.86405	4.767414
H	−2.239581	0.88643	5.800793
H	−4.01123	1.935325	−0.925806
C	−5.693081	3.142671	−0.314078
C	−3.549547	4.003472	−1.309112
H	−6.14464	3.130321	−1.318898
H	−6.200137	2.387134	0.303883
H	−5.886105	4.127398	0.139665
H	−3.963023	3.914847	−2.326167
H	−3.812573	5.005243	−0.935793
H	−2.456238	3.92975	−1.367719
H	−2.693694	0.490755	−1.952853
C	−1.79343	0.709983	−3.887955
C	−4.169085	0.010109	−3.442421
H	−2.039706	1.782001	−3.869793
H	−0.735924	0.595637	−3.62588
H	−1.937339	0.350749	−4.919736
H	−4.474563	1.059612	−3.58658
H	−4.233021	−0.506547	−4.413075
H	−4.880728	−0.46414	−2.751779
H	2.898571	−1.815408	−1.448757
C	4.956283	−2.241912	−1.948849

Table S18. *Cont.*

Atomtype	X Coordinates	Y Coordinates	Z Coordinates
C	3.100544	−3.927651	−1.782105
H	2.862248	−3.796525	−2.849186
H	3.858973	−4.723803	−1.70718
H	2.192171	−4.258427	−1.260641
H	4.788981	−2.242382	−3.038457
H	5.335946	−1.251646	−1.664254
H	5.730153	−2.992434	−1.720552
H	3.924656	0.800145	2.522617
C	3.518557	0.035568	4.489848
C	5.807731	0.28387	3.441707
H	3.614636	1.035223	4.942829
H	2.452825	−0.176872	4.326824
H	3.91057	−0.695235	5.214669
H	5.919448	1.248029	3.96364
H	6.236562	−0.50658	4.078585
H	6.390569	0.323361	2.509097
H	3.400816	0.093709	−2.510162
C	1.660986	−0.625001	−3.535305
C	3.657876	0.552599	−4.581465
H	4.112126	−0.426468	−4.802972
H	3.076222	0.866462	−5.462831
H	4.465635	1.284242	−4.424433
H	2.124095	−1.605933	−3.727721
H	1.02588	−0.710594	−2.641198
H	1.032303	−0.373582	−4.403962
H	2.637103	3.597811	0.637572
C	3.492574	5.359767	−0.257702
C	1.113632	5.118545	0.587773
H	1.423841	5.536129	1.558632
H	0.769875	5.956262	−0.03939
H	0.264614	4.441942	0.762804
H	3.771109	5.823156	0.702504
H	4.374776	4.839517	−0.662399
H	3.216475	6.160233	−0.962877

Table S19. Cartesian geometry of transition state (+ 18.0 kcal/mol) in Figure S8 in Angstrom [Å].

Atomtype	X Coordinates	Y Coordinates	Z Coordinates
N	3.456641	0.987754	−0.44128
C	2.611132	−0.045507	−0.087569
N	3.394437	−1.184786	0.011827
C	4.695988	−0.852613	−0.321206
C	4.731674	0.473712	−0.612459
N	1.232089	0.027424	0.004623
Si	0.000041	−0.106157	1.336297
C	0.266511	1.253845	2.61645
C	3.074705	−2.520849	0.682158
C	2.951502	−2.225071	2.191129
C	3.204915	2.49957	−0.552146

Table S19. *Cont.*

Atomtype	X Coordinates	Y Coordinates	Z Coordinates
C	2.226057	2.947773	0.54048
N	−1.199252	−0.040819	−0.015398
Si	0.021704	0.022158	−1.352509
C	−0.467038	1.434394	−2.502771
C	−2.56915	0.079743	−0.133993
N	−3.445011	−0.907264	−0.540736
C	−4.707538	−0.348707	−0.66864
C	−4.627126	0.962892	−0.328153
N	−3.311057	1.241744	0.002747
C	−3.314253	−2.440539	−0.567575
C	−3.761476	−2.930894	0.825001
C	−2.960275	2.529633	0.752379
C	−2.961366	2.154295	2.248252
C	−0.34135	−1.668206	2.342527
C	−1.629938	3.125561	0.264965
C	−4.05151	3.595091	0.503741
C	−4.253212	−2.997041	−1.666077
C	−1.895687	−2.912684	−0.884198
C	0.453603	−1.375216	−2.539994
C	1.822049	−3.154462	0.061058
C	4.239513	−3.513224	0.473182
C	4.542665	3.246518	−0.340296
C	2.655582	2.812882	−1.955251
H	−5.568018	−0.921726	−0.981265
H	−5.406433	1.710208	−0.299585
H	5.570453	1.081683	−0.916949
H	5.501353	−1.571999	−0.320878
H	−0.254066	−1.405717	−3.384306
H	1.439086	−1.11356	−2.969247
H	0.534953	−2.376184	−2.108033
H	−1.488667	1.16877	−2.83788
H	−0.528053	2.439978	−2.073548
H	0.16717	1.458673	−3.401695
H	−1.405677	−1.5978	2.632711
H	−0.204575	−2.626067	1.826795
H	0.236313	−1.690464	3.278597
H	0.078386	2.279972	2.281213
H	−0.335329	1.062214	3.518257
H	1.326565	1.194642	2.922263
H	−1.346156	3.934205	0.95383
H	−0.821252	2.395546	0.232561
H	−1.75711	3.562142	−0.735748
H	−2.678417	3.030533	2.849433
H	−3.968479	1.833109	2.552455
H	−2.259604	1.341289	2.466226
H	−3.693986	4.539871	0.93625
H	−4.222805	3.755028	−0.571039
H	−5.000507	3.34859	1.00026

Table S19. *Cont.*

Atomtype	X Coordinates	Y Coordinates	Z Coordinates
H	−1.15956	−2.524347	−0.177968
H	−1.895493	−4.010039	−0.816106
H	−1.617827	−2.640888	−1.908507
H	−3.793981	−4.030373	0.820848
H	−3.056923	−2.60649	1.604164
H	−4.76542	−2.555582	1.070682
H	−4.012479	−4.059447	−1.81
H	−5.314943	−2.946022	−1.386426
H	−4.101041	−2.47508	−2.622515
H	2.090302	4.034964	0.448693
H	1.254599	2.46616	0.417958
H	2.619072	2.730444	1.544378
H	2.740832	3.895084	−2.135577
H	3.224948	2.289008	−2.736776
H	1.593363	2.557979	−2.017514
H	4.306993	4.316439	−0.260585
H	5.043437	2.933149	0.587188
H	5.229765	3.130826	−1.190627
H	3.923798	−4.478648	0.89249
H	4.460682	−3.661866	−0.594074
H	5.151356	−3.208112	1.005638
H	1.551984	−4.041064	0.652424
H	0.973361	−2.471905	0.044247
H	2.036902	−3.479998	−0.966496
H	2.624495	−3.13165	2.72049
H	3.928723	−1.919027	2.592757
H	2.232689	−1.424196	2.399063

Table S20. Cartesian geometry of product (−18.9 kcal/mol) in Figure S8 in Angstrom [Å].

Atomtype	X Coordinates	Y Coordinates	Z Coordinates
C	4.640825	−0.824658	0.619754
N	3.357276	−1.2169	0.281087
C	2.597076	−0.083651	0.034334
N	3.447852	0.996954	0.173722
C	4.691575	0.531543	0.567257
C	3.050106	−2.663935	−0.113054
C	3.342747	2.474631	−0.235756
N	1.21825	−0.042391	−0.080174
Si	−0.082026	−0.225551	−1.314638
C	−0.219293	1.035224	−2.705395
Si	0.081797	0.225437	1.315023
C	0.324443	1.881465	2.181035
N	−1.218414	0.043153	0.08044
C	−2.597256	0.083595	−0.033746
N	−3.357681	1.215956	−0.283755
C	−4.64133	0.822469	−0.620576
C	−4.691923	−0.533561	−0.563652

Table S20. *Cont.*

Atomtype	X Coordinates	Y Coordinates	Z Coordinates
N	−3.447951	−0.997558	−0.169357
C	−3.050781	2.66451	0.105216
C	−3.341763	−2.474396	0.242857
C	0.224509	−1.036611	2.704116
C	−0.329911	−1.881756	−2.178865
H	−5.518564	−1.204635	−0.750738
H	−5.42272	1.526959	−0.865385
H	5.518227	1.20198	0.756586
H	5.421967	−1.530079	0.862682
H	0.320547	−1.948051	−3.065918
H	−0.177737	−2.786629	−1.578416
H	−1.363777	−1.887103	−2.563396
H	−0.147671	2.085233	−2.40983
H	0.549732	0.834386	−3.470978
H	−1.195357	0.88327	−3.199863
H	−0.542482	−0.838271	3.472332
H	0.154112	−2.0864	2.407426
H	1.201831	−0.883279	3.195729
H	0.165796	2.786528	1.58253
H	−0.324306	1.942529	3.069762
H	1.359148	1.891744	2.563075
C	−4.192625	3.589416	−0.363616
C	−2.986972	2.684078	1.645086
C	−1.753801	3.144423	−0.556979
H	−1.532379	4.157217	−0.190394
H	−0.913374	2.492123	−0.320282
H	−1.876862	3.191781	−1.647885
H	−2.722381	3.694053	1.990538
H	−3.966661	2.41659	2.067852
H	−2.237823	1.978533	2.022059
H	−3.892525	4.621258	−0.134742
H	−4.357108	3.516386	−1.449015
H	−5.133515	3.394056	0.170324
C	−4.472175	−2.739365	1.269633
C	−3.54182	−3.351202	−1.006786
C	−2.012255	−2.77196	0.931862
H	−4.348041	−3.761347	1.655308
H	−4.407671	−2.036163	2.113119
H	−5.473138	−2.673827	0.82287
H	−3.582532	−4.402807	−0.687619
H	−4.487567	−3.12004	−1.517945
H	−2.715999	−3.240251	−1.720264
H	−2.005056	−3.839415	1.193636
H	−1.155224	−2.564762	0.289752
H	−1.926081	−2.192077	1.857485
C	4.472254	2.739961	−1.263449
C	2.0127	2.774912	−0.922598
C	3.545263	3.349193	1.015054

Table S20. *Cont.*

Atomtype	X Coordinates	Y Coordinates	Z Coordinates
H	2.006286	3.84289	−1.182239
H	1.924954	2.196882	−1.849183
H	1.156414	2.567325	−0.279647
H	4.349623	3.763143	−1.646427
H	5.473685	2.671176	−0.818231
H	4.405121	2.038956	−2.108563
H	3.585773	4.401303	0.69756
H	2.720559	3.237447	1.729718
H	4.491755	3.116949	1.524331
C	4.192572	−3.590593	0.350713
C	1.753984	−3.14623	0.549112
C	2.984489	−2.67746	−1.652923
H	3.892581	−4.621479	0.117426
H	5.132989	−3.392485	−0.183049
H	4.357863	−3.522436	1.436303
H	2.717904	−3.68558	−2.002211
H	2.235967	−1.969224	−2.02607
H	3.964024	−2.409548	−2.075758
H	1.531435	−4.15732	0.178536
H	1.878712	−3.198223	1.639633
H	0.9136	−2.492418	0.31655

References

1. Sheldrick, G.M. *SHELX−97 Program for Crystal Structure Determination*; Universität Göttingen: Göttingen, Germany, 1997.
2. Frisch, M.J.; Trucks, G.W.; Schlegel, H.B.; Scuseria, G.E.; Robb, M.A.; Cheeseman, J.R.; Scalmani, G.; Barone, V.; Mennucci, B.; Petersson, G.A.; et al. *Gaussian 09, Revision B.01*; Gaussian, Inc.: Wallingford, CT, USA, 2009.
3. Lee, C.; Yang, W.; Parr, R.G. Development of the Colle-Salvetti correlation-energy formula into a functional of the electron density. *Phys. Rev. B* **1988**, *37*, 785–789.
4. Becke, A.D. Density-functional thermochemistry. III. The role of exact exchange. *J. Chem. Phys.* **1993**, *98*, 5648.
5. Hay, P.J.; Wadt, W.R. Ab initio effective core potentials for molecular calculations. Potentials for K to Au including the outermost core orbitals. *J. Chem. Phys.* **1985**, *82*, 299–310.
6. Petersson, G.A.; Al-Laham, M.A. A complete basis set model chemistry. II. Open-shell systems and the total energies of the first-row atoms. *J. Chem. Phys.* **1991**, *94*, 6081–6090.
7. Blaudeau, J.-P.; McGrath, M.P.; Curtiss, L.A.; Radom, L. Extension of Gaussian-2 (G2) theory to molecules containing third-row atoms K and Ca. *J. Chem. Phys.* **1997**, *107*, 5016–5021.
8. Weigend, F.; Ahlrichs, R. Balanced basis sets of split valence, triple zeta valence and quadruple zeta valence quality for H to Rn: Design and assessment of accuracy. *Phys. Chem. Chem. Phys.* **2005**, *7*, 3297–3305.
9. Weigend, F. Accurate Coulomb-fitting basis sets for H to Rn. *Phys. Chem. Chem. Phys.* **2006**, *8*, 1057–1065.
